# Neurodevelopmental and Psychosocial Outcomes in Adolescence of Children with Early Diagnoses of ADHD, Autism, Dyscalculia and Dyslexia

**DOI:** 10.1007/s10802-025-01377-z

**Published:** 2025-11-05

**Authors:** Yufei Cai, Joni Holmes, Giorgia Michelini, Thalia C. Eley, Susan E. Gathercole

**Affiliations:** 1 Department of Psychiatry, University of Cambridge, Cambridge, England United Kingdom; 2 School of Psychology, University of East Anglia, Norwich, England United Kingdom; 3 School of Biological and Behavioural Sciences, Queen Mary University of London, London, England United Kingdom; 4 Institute of Psychiatry, King’s College London, London, England United Kingdom

**Keywords:** ADHD, Autism, Dyslexia, Dyscalculia, Neurodiverse conditions

## Abstract

The study examined neurodevelopmental and psychosocial outcomes in adolescents aged 12 and 16 with childhood diagnoses of ADHD, autism, dyscalculia, or dyslexia. Participants were drawn from the Twins Early Development Study based on parent-reported diagnoses between ages 7 and 9 of ADHD (*n* = 54), autism (*n* = 50), dyscalculia (*n* = 282), and dyslexia (*n* = 695). A comparison group included 6,882 participants without neurodevelopmental diagnoses. Differences in ADHD and autistic traits, academic challenges, peer difficulties, and internalizing issues were explored between the comparison group and each neurodivergent group at ages 12 and 16. Across timepoints, neurodivergent groups showed distinct patterns of difficulties across domains relative to the comparison group. The ADHD group had higher levels of inattention and hyperactivity/impulsivity at 12 and 16, lower academic performance at 16, and elevated mental health challenges at 12. The autism group showed higher degrees of inattention, hyperactivity/impulsivity, autistic traits, and peer difficulties at 12 and 16. The dyscalculia group had challenges in all domains except for peer relationships at 12, with only mathematical underachievement persisting to 16. The dyslexia group showed difficulties in all domains at 12 with issues related to inattention, hyperactivity/impulsivity, academic achievement, and peer relationship persisting to 16. Whereas at age 12 the neurodivergent groups showed diagnosis-specific difficulties and broader neurodevelopmental and psychosocial challenges, by age 16 they were characterized by distinctive trajectories with persisting and resolved difficulties. These findings underscore the need for repeated, broad-based assessments to understand the changing needs of children with early neurodevelopmental diagnoses.

**Supplementary Information:**

The online version contains supplementary material available at 10.1007/s10802-025-01377-z.

Despite the distinctive diagnostic features defined by conventional psychiatric nosologies (American Psychiatric Association Division of Research, 2013; World Health Organization, 1992), many children diagnosed with neurodiverse conditions experience a broad and overlapping spectrum of challenges that can persist into adulthood. In this study, we examine the range and overlap of challenges experienced during early and middle adolescence by children diagnosed with one of four common neurodevelopmental conditions: attention deficit hyperactivity disorder (ADHD), characterized by developmentally inappropriate difficulties involving inattention and hyperactivity and / or impulsivity; autism, a condition associated with significant challenges in social interaction and communication, coupled with focused interests and repetitive patterns of behavior; dyscalculia, a form of specific learning disability (SLD) characterized by difficulties in understanding and applying arithmetic concepts; and dyslexia, an SLD characterized by difficulties in developing fluent reading. Irrespective of their primary diagnosis, children with these conditions often share characteristics of other undiagnosed neurodevelopmental conditions and commonly experience social and internalizing difficulties. Current understanding of the neurodevelopmental and psychosocial characteristics of ADHD, autism, dyscalculia and dyslexia is summarized below.

## ADHD

Approximately 20% of children diagnosed with ADHD meet the criteria for autism (Hollingdale et al., 2020), and core autistic traits including difficulties in social interaction, communication challenges, focused interests, and repetitive behaviors are commonly observed in children with ADHD (Craig et al., 2015; Green et al., 2015). Twin studies indicate that genetic factors account for most of the association between ADHD features and autistic traits, with minimal contributions of environmental factors (Pinto et al., 2016; Taylor et al., 2015). These findings indicate that the co-occurrence of ADHD and autistic traits is primarily driven by shared genetic mechanisms (Andersson et al., 2020).

Up to 45% of children with ADHD also meet criteria for SLD in reading or mathematics (DuPaul et al., 2013). Longitudinal analyses of population-based cohorts have shown that elevated ADHD features in early childhood are strongly associated with lower academic achievement in adolescence, with learning outcomes more significantly predicted by features of inattention than hyperactivity/impulsivity (Sayal et al., 2015; Tan et al., 2022). These findings align with proposals that successful learning requires strong attentional control to maintain focus and minimize distractions during academic tasks (Geary et al., 2020).

Difficulties in peer relations are widely reported in children with ADHD (Gardner & Gerdes, 2015). A possible explanation is that ADHD features contribute to poor social skills, which in turn impair the ability to form positive peer interactions (Murray-Close et al., 2010). Features of hyperactivity/impulsivity and inattention contribute differently to social difficulties. Children with a predominantly hyperactive ADHD presentation are more likely to experience high levels of peer rejection than those with a predominantly inattentive presentation (Thompson et al., 2023). Hyperactive/impulsive behaviors such as failing to take turns and initiating play disruptively or aggressively are linked to low peer acceptance and increased social isolation. In contrast, children with a predominantly inattentive presentation often struggle to build and maintain social connections (Ahmad et al., 2021). Elevated distractibility may interfere with picking up social cues and engaging in role-switching, making it harder to form and maintain friendships.

High rates of depression and anxiety are commonly reported in children with ADHD, with estimated comorbidity rates of 14% for depression and 18% for anxiety (Larson et al., 2011). One potential factor linking childhood ADHD to these internalizing challenges is peer relationship difficulties. A recent longitudinal study has shown that high levels of childhood ADHD features at age 7 significantly predicted increased depressive symptoms at age 17, an association partially accounted for by peer relationship difficulties as a mediator at age 16 (Powell et al., 2020). This indicates that features of inattention and hyperactivity/impulsivity may increase the risk of peer rejection and thereby contributing to higher rates of depression and anxiety.

## Autism

Elevated levels of inattention and hyperactivity/impulsivity are frequently reported in children with autism (Cervin, 2023; Lebeña et al., 2023), with approximately 38% meeting diagnostic criteria for ADHD (Rong et al., 2021). Around 20% of individuals with autism also have co-occurring SLD (Khachadourian et al., 2023). Educational outcomes among children with autism vary widely, from below to above average, with this variability largely attributed to differences in intellectual ability (Keen et al., 2015). Those with higher IQs typically meet or exceed academic expectations, whereas those with lower IQs often demonstrate academic underperformance in reading and mathematics (Kim et al., 2018).

Children with autism are four times more likely to face peer rejection and bullying than their neurotypical peers (Cresswell et al., 2019). It seems likely that these peer relationship difficulties arise from the social communication challenges that are fundamental to autism. They are more often rated by peers as undesirable social partners, less cooperative, and more likely to break social rules than their neurotypical peers (Billington et al., 2024). Such low peer acceptance may result from difficulties in interpreting social cues and understanding others’ intentions, leading to unintentional rule-breaking and misunderstandings.

Prevalence rates of depression and anxiety among individuals with autism range between 10% and 20% (Lai et al., 2019). Rodriguez et al. (2021) studied the bidirectional relationship between peer problems and symptoms of depression and anxiety in children and adolescents with autism over one year. They found that high levels of victimization at age 9 were significantly associated with increased depression and anxiety at age 10, but not vice versa. These findings suggest that peer relationship difficulties may contribute to subsequent internalizing challenges in children with autism.

## SLD in Reading and Mathematics

Within the broad category of diagnosed SLD, challenges in mathematics (dyscalculia) and reading (dyslexia) commonly co-occur, with reported comorbidity rates ranging from 11 to 70% (Moll et al., 2014). Both groups show reading and mathematics challenges from childhood that often persist into adolescence (Duff et al., 2022; Nelson & Powell, 2018).

Elevated levels of inattention and hyperactivity/impulsivity have been commonly reported in children with dyscalculia or dyslexia (Luoni et al., 2023; Willcutt & Petrill, 2023). Twin studies examining the relative influence of genetic and environmental factors have identified strong genetic contributions to the association between ADHD traits and achievement in reading and mathematics, with negligible environmental influence (Greven et al., 2014; Liu et al., 2019). Although autistic traits such as difficulties in social interaction and communication challenges, have also been reported in children with dyscalculia or dyslexia (Brimo et al., 2021; Morsanyi et al., 2018), research investigating the origins of these co-occurring features is currently limited.

Children with dyscalculia or dyslexia report higher incidences of bullying by peers compared to those without SLD (Weinreich et al., 2023). Difficulties with reading and mathematics in childhood have been longitudinally associated with greater peer relationship difficulties in adolescence (Turunen et al., 2017; Wakeman et al., 2023). These children frequently reported being teased by peers when asked to read aloud or solve mathematics problems in front of the class (Berchiatti et al., 2022; Wilmot et al., 2023), suggesting that bullying experiences may be directly related to their slow academic progress.

Both groups show higher levels of depression and anxiety symptoms than their neurotypical peers (Vieira et al., 2024). Some research suggests that receiving feedback related to reading or mathematics skills, including discouraging comments from teachers or consistently poor exam performance, may negatively affect their academic self-perception (Wilmot et al., 2023). This raises the possibility that losing confidence in their ability to succeed academically contributes to heightened anxiety and depression as they progress through their education.

## A Transdiagnostic Approach

The overlapping neurodevelopmental and psychosocial characteristics of children with different neurodevelopmental conditions combined with high variability in the profiles of individuals with the same diagnoses have led many researchers and clinicians to adopt a transdiagnostic approach rather than a diagnosis-centered perspective to understanding the specific challenges faced by neurodivergent individuals. Aligning with the influential RDoC framework and broader trends in adult psychopathology research (Dalgleish et al., 2020), this approach characterizes neurodivergent individuals based on their profiles across multiple dimensions of neurodevelopmental and psychopathological characteristics, rather than assigning discrete categorical labels. Individual profiles across these dimensions can be conceptualized as representing a spectrum of neurodevelopmental risk, which can guide assessment and tailor support to the needs of the individual child, either as an alternative or adjunct to standard diagnostic categories (Astle et al., 2022; Michelini et al., 2024).

The aim of the present study is to provide a broad transdiagnostic characterization of the profiles of individuals diagnosed with ADHD, autism, dyscalculia, or dyslexia by age 9 as they progress from early to middle adolescence. While the above review highlights extensive evidence of overlapping traits dimensions among these conditions, the current understanding of broad longitudinal outcomes remains limited by the paucity of direct comparisons across multiple neurodevelopmental conditions and traits dimensions. Available evidence is fragmented, assembled from studies usually with just one or two neurodivergent groups and employing a wide range of different age ranges, comparison groups, assessment methods, and analytic techniques. The scarcity of broad-based comparison of multiple neurodevelopmental conditions across domains makes it difficult to draw robust conclusions regarding the long-term impact of individual neurodevelopmental conditions on later neurodevelopmental and psychosocial outcomes.

This pre-registered study (https://osf.io/y7erv) addressed these limitations by directly comparing groups with early diagnoses of distinct neurodevelopmental conditions to a neurotypical group drawn from the same national population, using the same assessments of neurodevelopmental and psychosocial constructs from early to middle adolescence. Participants were drawn from the Twins Early Development Study (TEDS), a longitudinal population study in the United Kingdom (Lockhart et al., 2023). The groups included children diagnosed with ADHD, autism, dyscalculia, or dyslexia by age 9, and a comparison group without any reported neurodevelopmental conditions. At ages 12 and 16, neurodevelopmental features were assessed, in addition to two psychosocial domains: peer relationships and internalizing challenges, including depression and anxiety. Consistent with prior research, it was anticipated that all four neurodivergent groups would show higher levels of ADHD features and autistic traits, more peer relationship difficulties, and greater symptoms of anxiety and depression than their neurotypical peers. Lower academic achievement was predicted for the groups with ADHD, dyscalculia, and dyslexia. Given that variability in academic outcomes among individuals with autism has been linked to intellectual ability(Keen et al., 2015; Kim et al., 2018), no specific predictions were made for this group’s educational attainment. Strong predictions regarding the developmental timing of elevated symptom profiles could not be made due to the paucity of relevant research directly comparing neurodevelopmental and psychosocial outcomes across all four groups from early to middle adolescence.

## Method

### Participants

The sampling frame for this study comprised over 13,000 twins born in the United Kingdom between 1994 and 1996. Ethical approval for the study and the consent procedures was obtained from the relevant ethics committee (ref: PNM/09/10–104). Parents provided informed consent for each wave of data collection, and informed consent was obtained from the twins in adolescence. Details about the TEDS are available elsewhere (Lockhart et al., 2023).

### Measures and Procedure

Data collected at ages 7, 8, 9, 12, and 16 years were utilized in this study (https://www.teds.ac.uk/datadictionary/home.htm). Participants were first allocated to the ADHD, autism, dyscalculia, dyslexia, and comparison groups based on parent-reported diagnostic information between ages 7 and 9. The comparison group was matched for gender distribution and mean age with the neurodivergent groups utilizing the *MatchIt* R package. Individuals diagnosed with two or more of the above neurodevelopmental conditions were excluded due to small group sizes (see Table 1., Supplementary Material). The lower prevalence of multiple neurodevelopmental diagnoses may reflect the use of earlier versions of the DSM and ICD diagnostic frameworks, which did not explicitly recognize comorbidity (American Psychiatric Association Division of Research, 2013; World Health Organization, 1992).

In the United Kingdom, ADHD and autism diagnoses are based on assessments conducted by trained professionals in regional NHS clinics, consultant child psychologists or psychiatrists within NHS services, or qualified specialists in private practice. These evaluations adhere to established diagnostic criteria and involve clinical assessments in line with international standards (American Psychiatric Association Division of Research, 2013; World Health Organization, 1992).

SLD diagnoses including dyscalculia and dyslexia are established through assessments conducted by specialist teachers or educational psychologists trained in identifying these conditions. Assessments can be requested by schools through the Special Educational Needs Coordinator, conducted in private practice, or arranged by national and local dyslexia or dyscalculia organizations. In common with other population-based cohorts (e.g., Cervin, 2023; Mandy et al., 2022; May et al., 2021), the TEDS recorded parental reports of the diagnostic status of their child’s neurodevelopmental conditions without further clinical information. Due to the absence of detailed clinical assessment records, it is not possible to identify any potential overlap with the measures used in the TEDS protocol.

### ADHD and Autism Features

At 12 and 16 years the two primary diagnostic criteria for ADHD, inattention and hyperactivity/impulsivity, were evaluated using the Conners’ Parent Rating Scale–Revised (CPRS-R; Conners et al., 1998). Internal consistency for the CPRS-R ranges from 0.85 to 0.92. Test–retest reliability is 0.78 for the Inattention subscale and 0.71 for the Hyperactivity/Impulsivity subscale (Conners et al., 1998). These features were also assessed using the parent-reported Hyperactivity/Inattention subscale of the Strengths and Difficulties Questionnaire (SDQ; Goodman, 1997). Internal consistency is 0.78 and test–retest reliability is 0.84 for the Hyperactivity/Inattention subscale of the SDQ (Muris et al., 2003). Autistic traits were rated by parents using the Childhood Asperger Syndrome Test (CAST; Scott et al., 2002) at age 12 and the Autism Spectrum Quotient (AQ; Baron-Cohen et al., 2001) at age 16.

Internal consistency for the CAST ranges from 0.71 to 0.81 (Holmboe et al., 2014), and for the AQ, it is 0.67 (Baron-Cohen et al., 2001). Test–retest reliability is 0.83 for the CAST and 0.70 for the AQ (Baron-Cohen et al., 2001; Williams et al., 2006).

### Learning

At age 12, participants completed the Reading Fluency (Yes/No) Web Test (Woodcock, 2001), and the Number Game Web Test. Internal consistency for the Reading Fluency (Yes/No) Web Test ranges from 0.87 to 0.94 (Schrank & McGrew, 2001). The Number Game Web Test includes items drawn from National Foundation for Educational Research (NFER) booklets (Nfer-Nelson Publishing Co. Ltd, 1999), which are a range of assessments that reflect the mathematics curriculum requirements in the United Kingdom. No reliability estimates are available for the Number Game Web Test, as the TEDS team selected items from multiple NFER booklets.

At age 16, participants completed General Certificate of Secondary Education (GCSE) exams in English and Mathematics. The Mathematics assessments evaluated students’ abilities to apply mathematical concepts, reason logically, and solve problems. The English assessments measured reading comprehension, written expression, and the accurate use of grammar and spelling. Analyses were performed on the converted overall GCSE grades for each subject, based on an 8-point ordinal scale ranging from 4 (lowest grade, G) to 11 (highest grade, A*), as provided by the TEDS. Reliability estimates are not available for GCSE exams because they comprise multiple assessment components with differing weights and formats, making it difficult to compute a reliability index (Department for Education, 2013).

### Peer Relationship Difficulties

The Peer Problems subscale of the SDQ (Goodman, 1997) was completed by parents and adolescents to assess peer relationship difficulties at ages 12 and 16. This subscale focused on issues such as being alone, having few friends, and experiencing bullying. Internal consistency is 0.66 for the parent-report version and 0.54 for the self-report version; corresponding test–retest reliabilities are 0.91 and 0.83 (Muris et al., 2003).

### Internalizing Issues

At age 12, adolescents rated their emotional difficulties using the Emotional Problems subscale of the SDQ (Goodman, 1997), which includes items assessing worries, fears, nervousness, sadness, and somatic symptoms. The internal consistency of the Emotional Problems subscale of the SDQ is 0.71, and the test–retest reliability is 0.76 (Muris et al., 2003). They reported depressive symptoms using the Mood and Feelings Questionnaire (MFQ; Angold et al., 1995). The internal consistency of the MFQ is 0.95, and the test–retest reliability is 0.80 (Burleson Daviss et al., 2006; Sund et al., 2001).

At age 16, adolescents completed the Emotional Problems subscale of the SDQ (Goodman, 1997) and the Short Mood and Feelings Questionnaire (SMFQ; Angold et al., 1995)^1^. The internal consistency of the SMFQ is 0.84 (Rhew et al., 2010), and test–retest reliability ranges from 0.76 to 0.88 (Fernández-Martínez et al., 2020; Espada et al. 2024). Additionally, they completed the Children’s Anxiety Sensitivity Index (CASI; Silverman et al., 1991) to assess anxiety traits. The internal reliability of the CASI is 0.87 (Weems et al., 2010), and the test–retest reliability is 0.79 (Silverman et al., 1991).

### Analysis Plan

Statistical analysis was conducted using R version 4.3.1. The study was pre-registered before data access, and necessary adjustments in group selection and analytic methods were implemented to optimize data availability (S1, Supplementary Materials). Individuals in each group with less than 20% missing data at ages 12 and 16 on principle measures were included in the main analyses. Missing data were imputed separately by group and timepoint using the *MICE* R package.

Random-intercept mixed models (RIMM) were performed using the *lme4* R package. The analyses investigate cross-sectional differences in neurodiverse features of ADHD and autism, learning, peer relationship difficulties, and internalizing issues between the neurodivergent and comparison groups at ages 12 and 16. The comparison group was set as the reference level for each model. In line with the pre-registration, family variance was included as a random intercept to account for the non-independence of twin data. Bonferroni correction set the significance threshold at *p* <.0125 to adjust for multiple group comparisons. Power analyses using the *simr* R package indicated a 90% chance of detecting significant group differences (*α* = 0.05) for each planned RIMM comparison.

Longitudinal analyses compared symptom assessments between each neurodivergent group and the comparison group using the subset of repeated measures completed at both ages 12 and 16. In each model, the comparison group was set as the reference level. Trends for the comparison group reflect its own slope, while interaction terms between group and age indicate whether a neurodivergent group had a steeper or flatter slope across this period. The RIMM analyses tested whether the rate of change in repeated measures at ages 12 and 16 differed significantly between each neurodivergent group and the comparison group. These measures included the Hyperactivity/Inattention, Peer Problems, and Emotional Problems subscales of the SDQ (Goodman, 1997), and the Inattention and Hyperactivity/Impulsivity subscales of the CPRS-R (Conners et al., 1998). Assessments of autistic traits and academic outcomes were excluded due to substantive changes in test instruments. Family variance was included as the random intercept, and the significance threshold of *p* <.0125 was consistent with the main cross-sectional RIMM analysis.

Unplanned 2 × 2 chi-square (*χ²)* tests were conducted to determine whether the proportions of participants with clinically elevated ADHD and autistic traits differed significantly between the comparison group and each neurodivergent group at both timepoints. Comparisons were based on the number of children in each group exceeding a cut-off value corresponding to the mean plus two standard deviations from the aggregated dataset (*N* = 7963). ADHD features were assessed using the Inattention and Hyperactivity/Impulsivity subscales of the CPRS-R (Conners et al., 1998) at ages 12 and 16. Autistic traits were measured using the CAST (Scott et al., 2002) at age 12 and the AQ (Baron-Cohen et al., 2001) at age 16.

## Results

**Fig. 1:**
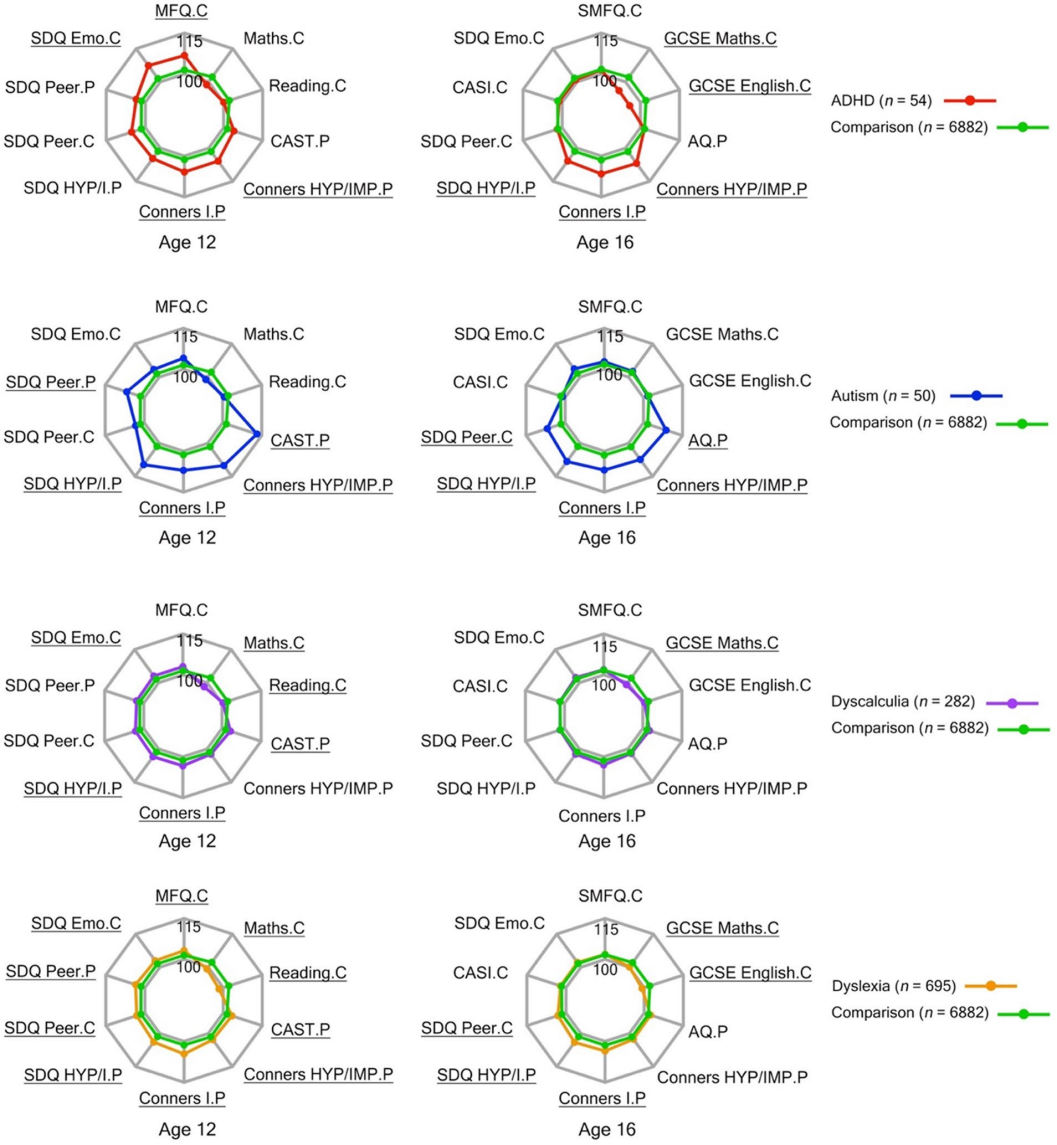
Profile of neurodivergent groups versus comparison group at ages 12 and 16. Note. Variables yielding significant differences (*p* <.0125) between each neurodiverse and the comparison group are underlined. Adjusted means and standard deviations from Tables 2 and 3 for each group were converted into standard scores (*M* = 100, *SD* = 15) for visualization. Norm-referenced scores were computed using the adjusted means and standard deviations of all groups. Higher scores indicate greater difficulties except for reading, mathematics, GCSE English, and GCSE Mathematics.

Table 1. presents descriptive statistics for the study groups. A one-way analysis of variance showed a significant difference in SES scores across groups [*F*_(4, 3415)_ = 5.49, *p* <.001]. The RIMM cross-sectional outcomes at ages 12 and 16 were unchanged when SES was added as a covariate (S2, Supplementary Materials). There were no significant differences in ethnicity across the groups [*χ²*_(4, *N* =7963)_ = 3.66, *p* =.455]. Tables 2 and 3 provide adjusted means of the principal measures at ages 12 and 16, derived from the RIMM cross-sectional analysis. Details of these analyses are summarized in S3 of the Supplementary Materials. Figure 1 illustrates the differences identified by RIMM cross-sectional analyses between each neurodivergent group and the comparison group at both time points. The following section describes neurodevelopmental and psychosocial differences at ages 12 and 16 between each neurodivergent group and the comparison group. Groups were selected based on parent-reported neurodevelopmental diagnoses between ages 7 and 9.

**Table 1.** Descriptive statistics for the study groups

	ADHD	Autism	Dyscalculia	Dyslexia	Comparison
*n*	54	50	282	695	6882
Age, y (*Mean* ± *SD*)	7.66 ± 0.62	7.42 ± 0.57	7.28 ± 0.71	7.29 ± 0.60	7.05 ± 0.25
Male, *n* (*%*)	31 (57%)	29 (58%)	119 (42%)	344 (49%)	2,999 (43%)
Ethnicity of the child					
White, *n* (*%*)	52 (96%)	48 (96%)	265 (94%)	669 (96%)	6498 (94%)
Other, *n* (*%*)	2 (4%)	2 (4%)	15 (5%)	26 (4%)	366 (5%)
Unavailable, *n* (*%*)	-	-	2 (1%)	-	18 (1%)
SES^2^ (*Mean*±*SD*)	−0.09 ± 0.98	0.32 ± 1.04	−0.00 ± 0.86	0.09 ± 1.08	0.32 ± 0.96

Note.^2^ The SES (socioeconomic status) index is composite measure derived from parental occupation and education levels, and the mother’s age at the birth of her first child (Lockhart et al., 2023)

**Table 2:** Adjusted means for all groups at age 12

	ADHD			Autism			Dyscalculia			Dyslexia			Comparison		
ADHD symptoms	*M*	*SD*	*g*	*M*	*SD*	*g*	*M*	*SD*	*g*	*M*	*SD*	*g*	*M*	*SD*	
SDQ HYP/I.P	3.24	2.26	0.25*	4.19	2.27	0.62**	3.01	2.25	0.16	3.08	2.32	0.19	2.61	2.56	
Conners I.P	7.05	4.95	0.34*	7.56	4.97	0.42*	5.95	4.91	0.15	6.53	5.11	0.25*	5.11	5.78	
Conners HYP/IMP.P	5.46	3.82	0.32*	7.00	3.85	0.63**	4.17	3.76	0.06	4.33	4.01	0.09	3.88	4.98	
Autism symptoms															
CAST.P	5.28	3.25	0.19	7.95	3.27	0.87***	5.07	3.22	0.13	5.11	3.37	0.14	4.56	3.90	
Learning															
Reading.C	56.91	12.99	−0.16	57.62	13.06	−0.11	57.32	12.85	−0.13	55.11	13.47	−0.28*	59.41	15.85	
Maths.C	65.20	13.50	−0.26*	65.41	13.56	−0.25*	64.61	13.38	−0.302*	65.81	13.97	−0.23*	69.41	16.01	
Peer relationship difficulties															
SDQ Peer.P	1.28	1.47	0.15	1.70	1.48	0.32*	1.18	1.45	0.09	1.29	1.52	0.16	1.02	1.74	
SDQ Peer.C	1.84	1.62	0.28*	1.58	1.62	0.14	1.55	1.61	0.12	1.55	1.66	0.12	1.33	1.83	
Internalizing issues															
MFQ.C	3.70	3.34	0.39*	2.92	3.36	0.19	2.62	3.45	0.11	2.67	3.33	0.12	2.20	3.82	
SDQ Emo.C	3.16	2.15	0.44*	2.48	2.15	0.15	2.41	2.19	0.12	2.50	2.15	0.16	2.14	2.32	

Note. Hedges’ *g* is a measure of effect size (small*: >0.2 and < 0.5, medium**: >0.5 and < 0.8, large***: >0.8) (Nakagawa & Cuthill, 2007), quantifying the magnitude of difference between each neurodivergent group and the comparison group. SDQ HYP/I.P = Strength and Difficulties Questionnaire Hyperactivity/Inattention subscale (parent-completed), Conners I.P = Conners Inattention subscale (parent-completed), Conners HYP/IMP.P = Conners Hyperactivity/Impulsivity subscale (parent-completed), CAST.P = Childhood Asperger Syndrome Test (parent-completed), Reading.C = Yes/No web test (child-completed), Maths.C = Mathematics Web Test (child-completed). SDQ Peer.P = Strength and Difficulties Questionnaire Peer problems subscale (parent-completed), SDQ Peer.C = Strength and Difficulties Questionnaire Peer problems subscale (child-completed), MFQ.C = Mood and Feeling Questionnaire (child-completed), SDQ Emo.C = Strength and Difficulties Questionnaire Emotion subscale (child-completed). AQ.P = Autism Spectrum Quotient (parent-completed), GCSE English.C = General Certificate of Secondary Education English subject test (child-completed), GCSE Maths.C = General Certificate of Secondary Education Mathematics subject test (child-completed), SMFQ.P = Short Mood and Feeling Questionnaire (child-completed), CASI.C = Children’s Anxiety Sensitivity Index (child-completed)

**Table 3:** Adjusted means for all groups at age 16

	ADHD			Autism			Dyscalculia			Dyslexia			Comparison	
ADHD symptoms	*M*	*SD*	*g*	*M*	*SD*	*g*	*M*	*SD*	*g*	*M*	*SD*	*g*	*M*	*SD*
SDQ HYP/I.P	2.86	1.94	0.33*	3.26	1.95	0.51**	2.29	1.93	0.07	2.56	2.00	0.19	2.14	2.22
Conners I.P	6.03	4.74	0.38*	6.18	4.76	0.41*	4.53	4.70	0.11	4.78	4.90	0.16	3.91	5.58
Conners HYP/IMP.P	3.89	3.15	0.39*	4.02	3.17	0.42*	2.46	3.10	0.03	2.62	3.29	0.07	2.33	3.99
Autism symptoms														
AQ.P	23.61	9.93	−0.01	30.81	10.01	0.52**	24.61	9.71	0.07	24.50	10.47	0.06	23.72	13.69
Learning														
GCSE English.C	8.38	1.16	−0.46*	9.02	1.16	−0.03	8.90	1.14	−0.11	8.74	1.21	−0.22*	9.06	1.49
GCSE Maths.C	8.34	1.33	−0.45*	9.19	1.34	0.03	8.74	1.29	−0.22*	8.87	1.40	−0.15	9.13	1.77
Peer relationship difficulties														
SDQ Peer.C	1.54	1.53	0.02	2.18	1.53	0.39*	1.53	1.53	0.01	1.70	1.57	0.11	1.51	1.70
Internalizing issues														
SMFQ.C	3.29	4.52	−0.06	4.33	4.53	0.15	3.81	4.64	0.05	3.76	4.52	0.04	3.58	5.06
SDQ Emo.C	2.68	2.29	−0.02	2.92	2.29	0.08	2.76	2.35	0.01	2.75	2.28	0.01	2.73	2.49
CASI.C	7.52	5.95	−0.04	7.42	5.96	−0.05	7.93	2.11	0.01	8.15	5.95	0.03	7.88	9.25

Note. See Table 2

### ADHD

At 12 years, the ADHD group had significantly higher levels of parent-reported inattention and hyperactivity/impulsivity than the comparison group. There were no significant differences between the ADHD and comparison groups in parent-reported autistic traits, self-reported academic tests, or peer relationship difficulties reported by both parents and adolescents. The ADHD group had significantly higher levels of self-reported emotional challenges and depressive symptoms than the comparison group.

At 16 years, the ADHD group had significantly elevated levels of parent-reported inattention and hyperactivity/impulsivity compared to the comparison group. The ADHD group had significantly lower self-reported GCSE scores than the comparison group in both reading and mathematics. No significant differences were found in parent-reported autistic traits, self-reported peer relationship challenges, or self-reported internalizing issues between the ADHD and comparison groups.

### Autism

At 12 years, parent ratings of inattention, hyperactivity/impulsivity, and autistic traits were higher for the autism group than the comparison group. The autism group also showed significantly more parent-reported peer relationship problems than the comparison group, although corresponding self-reports did not differ between these two groups. The autism group’s performance on self-completed reading and mathematics tests did not significantly differ from the comparison group. There were no significant differences in self-reported internalizing issues between the autism group and the comparison group.

At 16 years, the autism group exhibited higher levels of parent-reported inattention, hyperactivity/impulsivity, and autistic traits than the comparison group. The autism group had greater self-reported peer relationship difficulties than the comparison group, with no significant differences in self-reported GCSE performance or internalizing challenges.

### Dyscalculia

At 12 years, the dyscalculia group had higher levels of parent-reported inattention, hyperactivity/impulsivity, and autistic traits than the comparison group. They also had lower scores on self-completed mathematics and reading tests. The dyscalculia and comparison groups did not significantly differ on either parent- or self-reported peer relationship difficulties. Self-reported emotional challenges were significantly higher in the dyscalculia group compared to the comparison group.

At 16 years, no significant differences were observed between the dyscalculia and comparison groups in parent-reported ADHD and autistic traits, self-reported peer relationship difficulties, internalizing issues, or GCSE English exam performance. Self-reported GCSE Mathematics scores were significantly lower in the dyscalculia than the comparison group.

### Dyslexia

At 12 years, the dyslexia group had higher levels of parent-reported inattention, hyperactivity/impulsivity, and autistic traits than the comparison group. They had lower scores on self-completed reading and mathematics tests. The dyslexia group had greater self- and parent-reported peer difficulties and higher self-reported emotional and depressive symptoms than the comparison group.

At 16 years, the dyslexia group had higher levels of parent-reported inattention and hyperactivity/impulsivity than comparison group, while parent-reported autistic traits did not differ significantly between the two groups. The dyslexia group reported greater self-reported peer relationship difficulties and scored lower than the comparison group on self-reported GCSE Mathematics and English assessments. Self-reported internalizing issues did not differ between the two groups.

### Longitudinal Analyses

Longitudinal analyses examined the interaction between group status and age on repeated measures at ages 12 and 16 (S4, Supplementary Materials). Across the three ADHD feature measures, the comparison group showed decreasing scores between ages 12 and 16, representing reductions of features across time. The dyscalculia group showed a significantly faster decline in scores on the Hyperactivity/Inattention subscale of the SDQ, and the dyslexia group had a significantly steeper decrease in scores on the Inattention subscale of the CPRS-R. Both the autism and dyslexia groups showed significantly greater decreases in scores on the Hyperactivity/Impulsivity subscale of the CPRS-R. Scores on the Peer Problems and Emotional Problems subscales of the SDQ increased between ages 12 and 16 for the comparison group, reflecting a pattern of growing difficulties with age. In contrast, the dyscalculia group showed a significantly faster decline in scores on the Peer Problems subscale of the SDQ, and the ADHD, dyscalculia, and dyslexia groups all exhibited significantly steeper declines in scores on the Emotional Problems subscale of the SDQ over time.

### Clinically Elevated Neurodiverse Features

*χ²* tests compared each neurodivergent group with the comparison group at both timepoints on the proportions of individuals scoring above the clinical cutoff. Results are reported in S5 of the Supplementary Materials.

## Discussion

Drawing on data from a large population cohort, this study directly compared adolescents diagnosed with ADHD, autism, dyscalculia, or dyslexia by age 9 to a group with no neurodevelopmental conditions. It examined whether the four neurodivergent groups showed elevated levels of parent-reported ADHD and autistic traits, self-reported academic underachievement, parent- and self-reported peer difficulties, and self-reported internalizing challenges at ages 12 and 16, relative to the comparison group.

### Profiles of the Neurodivergent Groups

Adolescents with ADHD exhibited persistently elevated levels of parent-reported inattention and hyperactivity/impulsivity at ages 12 and 16, reinforcing evidence that childhood ADHD is a developmentally stable condition (Franke et al., 2018). Contrary to previous findings reporting elevated autistic traits in the ADHD group (Craig et al., 2015; Green et al., 2015), the current study found no significant group-level differences in parent-reported autistic traits at ages 12 and 16. However, at age 12, 19% of adolescents with ADHD exhibited clinically significant autistic traits, whereas the proportion was 4% in the comparison group (S5, Supplementary Materials). This outcome is consistent with evidence that 19% of children and adolescents with ADHD in community samples meet the diagnostic threshold for autism (Hollingdale et al., 2020). By age 16, the proportion of adolescents with elevated parent-reported autistic traits had declined to 4%, matching the level of the comparison group. This outcome reinforces reports by Fossum et al. (2021) of declining autistic traits in individuals with ADHD to non-clinical levels between ages 12 and 22. In focusing on the period from 12 to 16 years, the present study helps bridge the gap between early and middle adolescence. Findings indicate that elevated parent-reported autistic traits in early adolescence will have resolved by the end of secondary school for many young people with ADHD. Self-reported academic difficulties in reading and mathematics were not detectable in the ADHD group until 16 years, consistent with longitudinal research linking childhood ADHD features specifically to later academic underperformance in adolescence (Sayal et al., 2015). One possible explanation for the increasing risk of declining academic attainment in the later years of compulsory education is that the impact of attentional difficulties increases as academic demands become more intense and complex.

Although the ADHD group showed consistently high levels of parent-reported inattention and hyperactivity/impulsivity, they did not demonstrate increased peer relationship difficulties based on either parent or self-reports at ages 12 or 16. This outcome contrasts with previous studies linking early inattentive and hyperactive/impulsive behavior to later peer difficulties (Ahmad et al., 2021; Thompson et al., 2023). Elevated self-reported emotional challenges and depressive symptoms were observed in the ADHD group at age 12 but not at age 16. A previous longitudinal study by Powell et al. (2020) linked early childhood ADHD features to increased depressive symptoms in middle adolescence, with this association partially mediated by peer relationship difficulties at age 16. It is possible that adequate peer relationships in the present ADHD group provided a degree of resilience against developing internalizing difficulties.

The autism group exhibited persistent and complex difficulties at both ages 12 and 16. In addition to the expected elevated levels of parent-reported autistic traits at both time points (May et al., 2021), they also showed increased parent-reported inattentiveness and hyperactivity/impulsivity (Cervin, 2023; Lebeña et al., 2023). Their self-reported academic achievement did not differ from that of the comparison group. With parental reports indicating that none of the participants in the autism group had intellectual disabilities, this outcome is consistent with other evidence of robust academic performance in children with autism who have unimpaired cognitive abilities (Keen et al., 2015; Kim et al., 2018).

High levels of peer relationship difficulties were reported in the autism group at both time points, with parent-reported difficulties elevated at age 12 and self-reported difficulties noted at age 16. This outcome supports the view that social communication challenges make it particularly difficult for children with autism to maintain positive peer relationships (Cresswell et al., 2019). The autism group did not exhibit elevated levels of self-reported depression and anxiety at either time point, contrary to findings from Rodriguez et al. (2021) indicating that early peer relationship problems are associated with later depression and anxiety in children with autism. This discrepancy may be due to differences in the age periods examined in the two studies: whereas Rodriguez et al. (2021) investigated this relationship over a one-year period from ages 9 to 10 during middle childhood, the present study assessed outcomes over a four-year period spanning early to middle adolescence. It is possible that the negative impact of peer bullying on internalizing challenges dissipates over a longer timeframe.

At age 12, the dyscalculia group showed multiple challenges, including lower self-reported performance in reading and mathematics, increased parent-reported levels of inattention, hyperactivity/impulsivity, and autistic traits, as well as elevated self-reported emotional difficulties. These findings reinforce previous evidence linking early childhood mathematics difficulties to later elevations across multiple neurodevelopmental and psychological domains in early adolescence (Morsanyi et al., 2018; Nelson & Powell, 2018; Wakeman et al., 2023). At 16 years, only self-reported mathematical difficulties persisted, while all earlier challenges had apparently resolved. This finding replicates a longitudinal study by Auerbach et al. (2008), which followed 58 children diagnosed with dyscalculia at ages 10 to 11 and found that their attentional and emotional functioning remained within the age-typical range over the following six years. With a larger sample of nearly 300 individuals diagnosed with dyscalculia in childhood, the present study extends these findings, demonstrating that mild attentional and emotional difficulties were present at age 12 but diminished to levels comparable to the comparison group by age 16.

In marked contrast, the dyslexia group continued to experience difficulties through age 16. Reinforcing previous findings on educational underachievement in children with dyslexia (Duff et al., 2022; Nelson & Powell, 2018), this group consistently reported difficulties in reading and mathematics from childhood through adolescence. The dyslexia group also had elevated levels of parent-reported inattention and hyperactivity/impulsivity at both timepoints. Findings from a longitudinal study of children with dyslexia from preschool to early childhood showed that attentional challenges did not emerge until primary school (Jordan & Dyer, 2017). Our study extends this timeline by suggesting that once established, ADHD features in children with dyslexia can persist into the secondary school years of adolescence. Parent- and self-reported peer relationship challenges were evident in the dyslexia group at age 12, with self-reported difficulties persisting at age 16. These findings are consistent with prior evidence linking low academic skills to peer difficulties during adolescence (Turunen et al., 2017; Wilmot et al., 2023). We speculate that with increasing age, children may become more aware of individual differences in academic abilities. In secondary school, increasing academic demands may further exacerbate learning difficulties in adolescents with dyslexia, making these challenges more noticeable to peers and thereby increasing the likelihood of being bullied or teased. However, the elevated parent-reported autistic traits, self-reported emotional difficulties and depressive symptoms observed at age 12 were no longer evident at age 16.

### Implications

This study shows that core features of ADHD, autism, dyscalculia, and dyslexia, as well as internalizing challenges and peer functioning difficulties, are present from early to middle adolescence in many individuals with these neurodiverse conditions. The findings have direct implications for clinical practice in addressing the needs of young people during this developmental period. The changing needs of the neurodivergent groups point to the practical value of repeating assessments from the point of initial diagnosis at regular periods throughout adolescence. The variable needs across multiple domains highlight the value of broad-based assessments that bridge neurodevelopmental and psychosocial domains to capture the areas of challenge currently faced by the individual. This approach has two key merits. First, it helps tailor interventions to the individual’s current needs. Second, by identifying areas of strength, it can help compensate for other difficulties and potentially prevent the escalation of more severe and complex challenges over time (Astle et al., 2022).

The distinctive profiles of the four neurodivergent groups at 12 and 16 years provide a substantial basis for guiding expectations of practitioners and their families concerning the likely future impacts of early diagnoses of ADHD, autism, dyscalculia, and dyslexia (Michelini et al., 2024). Of the four groups, the autism group exhibited the highest degree of neurodevelopmental risk, with parent-reported features of autism and ADHD, and elevated peer relationship difficulties reported by either parents or self, extending through to the middle adolescent years. A positive outcome was that the self-reported academic achievement of the autism group was at an age-appropriate level. Children with dyslexia and those with ADHD were both located at an intermediate level of neurodevelopmental risk spectrum, with distinctive patterns of challenges across development. The dyslexia group had high levels of parent-reported inattention, hyperactivity/impulsivity, as well as self-reported academic struggles and peer relationship difficulties at both 12 and 16 years. Self-reported internalizing issues and parent-reported autistic traits were elevated at age 12 but these declined to levels comparable to the comparison group by age 16. In contrast, the ADHD group displayed persistently high levels of parent-reported inattention and hyperactivity/impulsivity at both ages, while self-reported academic underachievement was only detectable at age 16. Self-reported emotional difficulties and depressive symptoms that were elevated at age 12 were no longer significantly present at age 16. The group of children with dyscalculia showed the lowest levels of enduring neurodevelopmental risk. Despite parent-reported neurodevelopmental and self-reported emotional challenges at age 12, by age 16 the only difficulty detected in the broad assessment analyzed in this study was the continued low level of self-reported mathematical attainment in the dyscalculia group.

Although the ADHD, dyscalculia, and dyslexia groups experienced elevated levels of parent-reported ADHD features and autistic traits, as well as self-reported psychosocial difficulties in early adolescence, the severity of these challenges diminished with age. In the ADHD group, the proportion of children with clinically high levels of parent-reported hyperactivity/impulsivity declined from 26% at age 12 to 19% at age 16 (corresponding rates for the comparison group were 4% and 3%). Approximately 10% of individuals in the dyscalculia and dyslexia groups exhibited elevated levels of parent-reported inattention, hyperactivity/impulsivity, and autistic traits at age 12, with fewer than 8% showing these difficulties at age 16. Both the dyscalculia and dyslexia groups showed steeper declines in parent-reported inattentive and hyperactive/impulsive features between ages 12 and 16 than the comparison group. A reduction in psychosocial challenges with increasing age was also observed in some cases. Self-reported emotional difficulties in the ADHD and dyslexia groups declined over time, and self-reported peer relationship difficulties and emotional problems diminished in children with dyscalculia. These outcomes provide a substantial basis for optimism: by age 16, the great majority of children with early childhood diagnoses of ADHD, dyscalculia, and dyslexia no longer exhibited clinically significant features of parent-reported ADHD or autism and experienced fewer self-reported psychosocial difficulties.

### Limitations

The study had several limitations related to data availability and sample composition. First, individuals with multiple neurodevelopmental condition diagnoses were excluded due to the low rates of reported co-occurrence and limited data on key measures at ages 12 and 16. It is acknowledged that excluding individuals with comorbid conditions from analyses inevitably leads to an underestimation of transdiagnostic traits across the broader neurodiverse population (Dalgleish et al., 2020; Michelini et al., 2024).

Second, group selection was based on parent-reported diagnoses without independent verification through access to assessment records. This limitation is common in many studies examining developmental outcomes in neurodivergent children using population cohorts (e.g., Mandy et al., 2022; May et al., 2021). Parental reports were also used to assess neurodiverse symptoms due to the limited availability of child and teacher reports at ages 12 and 16. It is inevitable that parental observations may not have fully captured the entire range of challenges and experiences of these individuals. Future studies incorporating data from multiple informants would provide a more comprehensive understanding of the lived experiences associated with these conditions.

Third, although uneven subgroup sizes are common in population-based cohorts (e.g., Cervin, 2023; Lebeña et al., 2023), the resulting differences in statistical power may have affected the sensitivity to detect group differences. For example, although a medium effect size was observed in the autism group and smaller effect sizes in the ADHD, dyscalculia, and dyslexia groups on the parent-reported Hyperactivity/Inattention subscale of the SDQ at age 12, the RIMM analysis identified significantly higher levels of inattentive/hyperactive behaviors in the autism, dyscalculia, and dyslexia groups than in the comparison group, but not in the ADHD group. These variations in sample size should be considered when interpreting null effects.

Finally, it was not possible to assess the potential drivers of developmental change across the trait dimensions underlying these neurodevelopmental conditions due to non-equivalent assessments of autistic traits, reading, and mathematics abilities at ages 12 and 16 (e.g., Taylor et al., 2013). Analysis of measures suitable for both age groups in future studies could offer valuable insights into the developmental processes underlying the unique and shared features of these four neurodiverse conditions from early to middle adolescence.

## Conclusions

This study examined neurodevelopmental and psychosocial outcomes at two timepoints from early to middle adolescence in individuals with childhood diagnoses of ADHD, autism, dyscalculia, or dyslexia. Each neurodivergent group exhibited consistently elevated levels of core features of their diagnosis from age 9 through 16. The groups were clearly distinguished by their varying degrees of broader neurodevelopmental and psychosocial challenges at different developmental stages. These outcomes reinforce concerns that relying exclusively on core diagnostic features fails to capture the broader challenges faced by neurodivergent adolescents. The present broad set of outcomes demonstrates that individuals with neurodevelopmental conditions face challenges that extend beyond diagnostic boundaries, although many of these difficulties often diminish to levels observed in the general population. The study highlights the importance of repeated and comprehensive assessments covering neurodevelopmental and psychosocial domains from the initial point of diagnosis through adolescence. The outcomes of such assessments will enable practitioners to tailor support strategies that address the changing spectrum of needs of neurodivergent individuals during the transition from the middle childhood years through to adulthood.

## Supplementary Information

Below is the link to the electronic supplementary material.

Supplementary Material 1 (DOCX 499 KB)

## Footnotes

1 The SMFQ included 13 items: 11 from the MFQ at age 12 and two non-overlapping items—one each from the Emotional Problems and Hyperactivity/Inattention subscales of the SDQ.
