# Supplementary material for: Neurodevelopmental and Psychosocial Outcomes in Adolescence of Children with Early Diagnoses of ADHD, Autism, Dyscalculia and Dyslexia
Source: Res Child Adolesc Psychopathol. 2025 Nov 5;53(12):2099–113. doi: 10.1007/s10802-025-01377-z (PMC12718279; doi:10.1007/s10802-025-01377-z)
Supplement: Supplementary file 1 — Supplementary Material 1 (DOCX 499 KB) [file 10802_2025_1377_MOESM1_ESM.docx]

Supplementary Information

S1: Pre-registration plans and deviations

1. Pre-registration plans
2. Group selection. Children will be assigned to groups based on parent-reported diagnoses (ADHD, autism, dyslexia, dyscalculia, or comparison group) at age 9.
3. Possible comorbid groups. If participant numbers permit, children with > 1 neurodevelopmental conditions of interest to the study (e.g., ADHD + autism, dyscalculia + dyslexia) will be allocated to groups reflecting their comorbid status. Power analyses will assess whether the group sizes have sufficient power.
4. Data independence. To ensure sampling independency, no more than one member of each twin pair will be selected for participation.
5. Statistical analysis. Group differences on key clinical and functional outcomes will be examined using multivariate analysis of variance (MANOVA) and analysis of variance (ANOVA).
6. Missing data. Selected participants will have no missing than 15% missing data. Missing values will be imputed.
7. Analyzed variables. Multi-informant data from parents, children, and teachers will be used for the main group comparisons at ages 12 and 16.
8. Deviations
9. Group selection. The selection criterion was changed to include parent-reported neurodevelopmental conditions diagnoses at ages 7, 8, or 9 years due to the limited data availability at 9 years.
10. No comorbid groups. Groups composed of participants with two neurodevelopmental conditions were not included due to small group sizes and limited data availability at ages 12 and 16 (see Table 1).
11. Inclusion of twin pairs. Both members of twin pairs meeting the group selection criteria were included to maximize data availability.
12. Statistical analysis. To account for family variance introduced by the changed selection criterion, random-intercept mixed-effect modeling was employed instead of ANOVAs and MANOVAs.
13. Missing data. The threshold for acceptable levels of missing data was raised to 20% to maximize data inclusion.
14. Analyzed variables. Parent-reported measures were used exclusively for the analyses due to the limited availability of teacher and child reports at ages 12 and 16. Child reports were utilized only when parent reports were unavailable.
15. Additional cross-sectional analysis controlling for SES at ages 12 and 16. Post-hoc Tukey HSD tests (*p* < .0125) indicated significantly higher SES in the comparison group than in the dyslexia group, with no other group differences. Additional RIMM analyses controlling for SES showed that SES differences did not impact the primary group comparison outcomes (Supplementary Information S2).
16. Longitudinal analysis for repeated measures at ages 12 and 16. A longitudinal RIMM were conducted on repeated measures collected at ages 12 and 16 to examine whether the rate of change differ between neurodivergent and comparison group. Measures of ADHD features, peer relationship difficulties, and emotional problems were included, while assessments of autistic traits and academic skills were excluded due to substantial changes in test instruments across timepoints (Supplementary Information S4).
17. Unplanned analysis for neurodiverse features. Chi-squared analyses (*χ²)* were conducted to explore the high levels of clinical features of ADHD and autism at ages 12 and 16 (Supplementary Information S5).

Table 1. Participant numbers for neurodivergent groups

|  | Age 7 | Age 8 | Age 9 | *N* participants meeting data missingness criteria at both 12 and 16 years |
| --- | --- | --- | --- | --- |
| Single NC groups (*n*) |  |  |  |  |
| ADHD | 20 | 34 | 0 | 54 |
| Autism | 30 | 20 | 0 | 50 |
| Dyslexia | 568 | 79 | 48 | 695 |
| Dyscalculia | 246 | n/a^1^ | 36 | 282 |
| Comorbid NC groups (*n*) |  |  |  |  |
| ADHD + Autism | 12 | 16 | 0 | 5 |
| ADHD + Dyslexia | 13 | 6 | 1 | 6 |
| ADHD + Dyscalculia | 10 | n/a^1^ | 11 | 0 |
| Autism + Dyslexia | 21 | 11 | 3 | 3 |
| Autism + Dyscalculia | 21 | n/a^1^ | 11 | 2 |
| Dyslexia + Dyscalculia | 424 | n/a^1^ | 138 | 0 |
| *N* of single NC and comorbid NC groups | 1365 | 166 | 248 | 1097 |
| *%* with comorbid conditions ^2^ |  |  |  |  |
| ADHD + Autism | 0.8 | 9.6 | 0 | - |
| ADHD + Dyslexia | 0.9 | 3.6 | 0.4 | - |
| ADHD + Dyscalculia | 0.7 | - | 4.4 | - |
| Autism + Dyslexia | 1.5 | 6.6 | 1.2 | - |
| Autism + Dyscalculia | 1.5 | - | 4.4 | - |
| Dyslexia + Dyscalculia | 31.0 | - | 55.6 | - |

*Notes:* NC = Neurodevelopmental conditions. ^1^Parent-reported diagnostic information for dyscalculia is unavailable at age 8. ^2^ *%* is calculated by dividing the *n* in each comorbid group by the *N* of single NC and comorbid NC groups at each age. Duplicate reports of diagnoses were removed.

S2. SES

Tukey HSD post-hoc tests comparing each neurodivergent group with the comparison group identified a significant difference only between the comparison and the dyslexia group (Table 2). To examine whether this group difference contributed to any of the reported differences on primary measures, a series of additional RIMM analyses, including SES as a covariate, were conducted on data at ages 12 and 16 (Tables 3 and 4).

|  | Mean Difference | 95% CI  Lower Bound | 95% CI  Upper Bound | *p*-value |
| --- | --- | --- | --- | --- |
| Comparison vs. ADHD | 0.41 | 0.33 | 1.14 | 0.555 |
| Comparison vs. Autism | 0.00 | 0.60 | 0.59 | 0.999 |
| Comparison vs. Dyscalculia | 0.32 | 0.63 | 0.02 | 0.031 |
| Comparison vs. Dyslexia | 0.23 | 0.41 | 0.05 | 0.004 |

Table 2.

*Note.* *p* < .0125 shown in bold

Table 3. Results of RIMM between neurodivergent and comparison groups by covarying SES at 12

| Strength and Difficulties Questionnaire hyperactivity/inattention subscale (parent-reported) | | | | | | | |
| --- | --- | --- | --- | --- | --- | --- | --- |
| Effects | Variable | *β* | *σ^2^* | *SE* | *SD* | *t* | *p* |
| Fixed | Intercept | 2.58 |  | 0.03 |  | 77.90 | **0.000** |
|  | ADHD | 0.71 |  | 0.35 |  | 2.02 | 0.043 |
|  | Autism | 1.48 |  | 0.32 |  | 4.59 | **0.000** |
|  | Dyscalculia | 0.40 |  | 0.14 |  | 2.84 | **0.004** |
|  | Dyslexia | 0.48 |  | 0.09 |  | 5.22 | **0.000** |
| Random intercept | Family variance |  | 1.75 |  | 1.32 |  |  |
|  | SES |  | 0.03 |  | 0.18 |  |  |
|  | Residual |  | 2.97 |  | 1.73 |  |  |
| Conners inattention subscale (parent-reported) | | | | | | | |
| Effects | Variable | *β* | *σ^2^* | *SE* | *SD* | *t* | *p* |
| Fixed | Intercept | 5.03 |  | 0.07 |  | 71.01 | **0.000** |
|  | ADHD | 2.68 |  | 0.76 |  | 3.51 | **0.000** |
|  | Autism | 2.42 |  | 0.70 |  | 3.46 | **0.000** |
|  | Dyscalculia | 0.74 |  | 0.30 |  | 2.42 | **0.012** |
|  | Dyslexia | 1.37 |  | 0.20 |  | 6.87 | **0.000** |
| Random intercept | Family variance |  | 9.67 |  | 3.11 |  |  |
|  | SES |  | 0.02 |  | 0.13 |  |  |
|  | Residual |  | 13.30 |  | 3.65 |  |  |
| Conners Hyperactivity/Impulsivity subscale (parent-reported) | | | | | | | |
| Effects | Variable | *β* | *σ^2^* | *SE* | *SD* | *t* | *p* |
| Fixed | Intercept | 3.82 |  | 0.06 |  | 61.13 | **0.000** |
|  | ADHD | 1.67 |  | 0.59 |  | 2.85 | **0.000** |
|  | Autism | 3.01 |  | 0.54 |  | 5.53 | **0.000** |
|  | Dyscalculia | 0.23 |  | 0.23 |  | 1.02 | 0.309 |
|  | Dyslexia | 0.43 |  | 0.15 |  | 2.80 | **0.005** |
| Random intercept | Family variance |  | 8.69 |  | 2.95 |  |  |
|  | SES |  | 0.08 |  | 0.28 |  |  |
|  | Residual |  | 6.82 |  | 2.61 |  |  |
| Childhood Asperger Syndrome Test (parent-reported) | | | | | | | |
| Effects | Variable | *β* | *σ^2^* | *SE* | *SD* | *t* | *p* |
| Fixed | Intercept | 4.53 |  | 0.05 |  | 89.38 | **0.000** |
|  | ADHD | 0.67 |  | 0.51 |  | 1.33 | 0.180 |
|  | Autism | 3.10 |  | 0.46 |  | 6.67 | **0.000** |
|  | Dyscalculia | 0.47 |  | 0.30 |  | 3.33 | **0.010** |
|  | Dyslexia | 0.52 |  | 0.13 |  | 3.92 | **0.000** |
| Random intercept | Family variance |  | 4.76 |  | 2.18 |  |  |
|  | SES |  | 0.09 |  | 0.30 |  |  |
|  | Residual |  | 5.55 |  | 2.36 |  |  |

*Note.* *p* < .0125 is shown in bold

Table 3. Cont

*Note.* *p* < .0125 is shown in bold

| Reading web test (child-completed) | |  |  |  |  |  |  |
| --- | --- | --- | --- | --- | --- | --- | --- |
| Effects | Variable | *β* | *σ^2^* | *SE* | *SD* | *t* | *p* |
| Fixed | Intercept | -59.50 |  | 0.24 |  | 252.03 | **0.000** |
|  | ADHD | -2.85 |  | 2.03 |  | -1.40 | 0.160 |
|  | Autism | -0.87 |  | 1.87 |  | -0.47 | 0.640 |
|  | Dyscalculia | -2.07 |  | 0.80 |  | -2.58 | **0.010** |
|  | Dyslexia | -4.49 |  | 0.53 |  | -8.46 | **0.000** |
| Random intercept | Family variance |  | 77.06 |  | 8.78 |  |  |
|  | SES |  | 6.21 |  | 2.49 |  |  |
|  | Residual |  | 88.52 |  | 9.41 |  |  |

Table 3. Cont.

| Maths web test (child-completed) | | | | | | | |
| --- | --- | --- | --- | --- | --- | --- | --- |
| Effects | Variable | *β* | *σ^2^* | *SE* | *SD* | *t* | *p* |
| Fixed | Intercept | 69.54 |  | 0.25 |  | 275.81 | **0.000** |
|  | ADHD | -3.64 |  | 2.09 |  | -1.74 | 0.082 |
|  | Autism | -3.01 |  | 1.92 |  | -1.57 | 0.116 |
|  | Dyscalculia | -4.58 |  | 0.83 |  | -5.53 | **0.000** |
|  | Dyslexia | -3.39 |  | 0.55 |  | -6.21 | **0.000** |
| Random intercept | Family variance |  | 70.97 |  | 8.42 |  |  |
|  | SES |  | 9.08 |  | 3.01 |  |  |
|  | Residual |  | 97.56 |  | 9.88 |  |  |
| Strength and Difficulties Questionnaire peer problems subscale (parent-reported) | | | | | | | |
| Effects | Variable | *β* | *σ^2^* | *SE* | *SD* | *t* | *p* |
| Fixed | Intercept | 0.99 |  | 0.02 |  | 45.28 | **0.000** |
|  | ADHD | 0.08 |  | 0.23 |  | 0.37 | 0.712 |
|  | Autism | 0.61 |  | 0.21 |  | 2.90 | **0.003** |
|  | Dyscalculia | 0.19 |  | 0.09 |  | 2.07 | 0.038 |
|  | Dyslexia | 0.26 |  | 0.06 |  | 4.40 | **0.000** |
| Random intercept | Family variance |  | 1.02 |  | 1.01 |  |  |
|  | SES |  | 0.00 |  | 0.03 |  |  |
|  | Residual |  | 1.12 |  | 1.06 |  |  |
| Strength and Difficulties Questionnaire peer problems subscale (child-reported) | | | | | | | |
| Effects | Variable | *β* | *σ^2^* | *SE* | *SD* | *t* | *p* |
| Fixed | Intercept | 1.31 |  | 0.02 |  | 58.21 | **0.000** |
|  | ADHD | 0.48 |  | 0.25 |  | 1.89 | 0.058 |
|  | Autism | 0.19 |  | 0.23 |  | 0.81 | 0.420 |
|  | Dyscalculia | 0.27 |  | 0.10 |  | 1.70 | 0.056 |
|  | Dyslexia | 0.22 |  | 0.07 |  | 3.30 | **0.000** |
| Random intercept | Family variance |  | 0.87 |  | 0.93 |  |  |
|  | SES |  | 0.00 |  | 0.00 |  |  |
|  | Residual |  | 1.57 |  | 1.25 |  |  |
| Mood and Feeling Questionnaire (child-reported) | | | | | |  |  |
| Effects | Variable | *β* | *σ^2^* | *SE* | *SD* | *t* | *p* |
| Fixed | Intercept | 2.17 |  | 0.05 |  | 46.00 | **0.000** |
|  | ADHD | 2.00 |  | 0.52 |  | 3.83 | **0.000** |
|  | Autism | 0.81 |  | 0.47 |  | 1.72 | 0.086 |
|  | Dyscalculia | 0.49 |  | 0.21 |  | 2.37 | 0.018 |
|  | Dyslexia | 0.40 |  | 0.14 |  | 2.97 | **0.003** |
| Random intercept | Family variance |  | 3.57 |  | 1.89 |  |  |
|  | SES |  | 0.03 |  | 0.18 |  |  |
|  | Residual |  | 6.66 |  | 2.58 |  |  |

*Note.* *p* < .0125 shown in bold

Table 3.

| Strength and Difficulties Questionnaire emotion subscale (child-reported) | | | | | | |  |
| --- | --- | --- | --- | --- | --- | --- | --- |
| Effects | Variable | *β* | *σ^2^* | *SE* | *SD* | *t* | *p* |
| Fixed | Intercept | 2.12 |  | 0.03 |  | 70.06 | **0.000** |
|  | ADHD | 1.25 |  | 0.34 |  | 3.71 | **0.000** |
|  | Autism | 0.38 |  | 0.31 |  | 1.23 | 0.217 |
|  | Dyscalculia | 0.36 |  | 0.13 |  | 2.64 | **0.008** |
|  | Dyslexia | 0.27 |  | 0.09 |  | 3.06 | **0.002** |
| Random intercept | Family variance |  | 1.30 |  | 1.14 |  |  |
|  | SES |  | 0.02 |  | 0.16 |  |  |
|  | Residual |  | 2.91 |  | 1.71 |  |  |

*Note.* *p* < .0125 shown in bold

Table 4. Results of RIMM between neurodivergent and comparison groups by covarying SES at 16

| Strength and Difficulties Questionnaire hyperactivity/inattention subscale (parent-reported) | | | | | | | |
| --- | --- | --- | --- | --- | --- | --- | --- |
| Effects | Variable | *β* | *σ^2^* | *SE* | *SD* | *t* | *p* |
| Fixed | Intercept | 2.11 |  | 0.03 |  | 72.10 | **0.000** |
|  | ADHD | 0.76 |  | 0.30 |  | 2.53 | **0.011** |
|  | Autism | 1.04 |  | 0.28 |  | 3.77 | **0.001** |
|  | Dyscalculia | 0.19 |  | 0.12 |  | 1.55 | 0.121 |
|  | Dyslexia | 0.41 |  | 0.08 |  | 5.14 | **0.000** |
| Random intercept | Family variance |  | 1.31 |  | 1.14 |  |  |
|  | SES |  | 0.04 |  | 0.20 |  |  |
|  | Residual |  | 2.16 |  | 1.47 |  |  |
| Conners inattention subscale (parent-reported) | | | | | | | |
| Effects | Variable | *β* | *σ^2^* | *SE* | *SD* | *t* | *p* |
| Fixed | Intercept | 3.83 |  | 0.07 |  | 54.58 | **0.000** |
|  | ADHD | 2.37 |  | 0.73 |  | 3.24 | **0.001** |
|  | Autism | 2.18 |  | 0.67 |  | 3.25 | **0.001** |
|  | Dyscalculia | 0.64 |  | 0.29 |  | 2.22 | 0.026 |
|  | Dyslexia | 0.87 |  | 0.19 |  | 4.56 | **0.000** |
| Random intercept | Family variance |  | 9.09 |  | 3.01 |  |  |
|  | SES |  | 0.10 |  | 0.32 |  |  |
|  | Residual |  | 12.06 |  | 3.47 |  |  |
| Conners Hyperactivity/Impulsivity subscale (parent-reported) | | | | | | | |
| Effects | Variable | *β* | *σ^2^* | *SE* | *SD* | *t* | *p* |
| Fixed | Intercept | 2.29 |  | 0.06 |  | 41.24 | **0.000** |
|  | ADHD | 1.57 |  | 0.48 |  | 3.26 | **0.001** |
|  | Autism | 1.59 |  | 0.44 |  | 3.57 | **0.000** |
|  | Dyscalculia | 0.15 |  | 0.19 |  | 0.81 | 0.417 |
|  | Dyslexia | 0.27 |  | 0.13 |  | 2.14 | 0.032 |
| Random intercept | Family variance |  | 5.03 |  | 2.24 |  |  |
|  | SES |  | 0.27 |  | 0.52 |  |  |
|  | Residual |  | 4.78 |  | 2.19 |  |  |
| Autism Spectrum Quotient(parent-reported) | | | | | | | |
| Effects | Variable | *β* | *σ^2^* | *SE* | *SD* | *t* | *p* |
| Fixed | Intercept | 23.59 |  | 0.17 |  | 139.23 | **0.000** |
|  | ADHD | 0.19 |  | 1.53 |  | 0.12 | 0.901 |
|  | Autism | 5.80 |  | 1.43 |  | 4.06 | **0.000** |
|  | Dyscalculia | 0.93 |  | 0.60 |  | 1.55 | 0.121 |
|  | Dyslexia | 0.84 |  | 0.40 |  | 2.08 | 0.037 |
| Random intercept | Family variance |  | 0.74 |  | 8.58 |  |  |
|  | SES |  | 0.00 |  | 0.00 |  |  |
|  | Residual |  | 0.44 |  | 6.62 |  |  |

*Note.* *p* < .0125 shown in bold

Table 4. Cont.

| GCSE english subject test (child-reported) | | |  |  |  |  |  |
| --- | --- | --- | --- | --- | --- | --- | --- |
| Effects | Variable | *β* | *σ^2^* | *SE* | *SD* | *t* | *p* |
| Fixed | Intercept | 9.05 |  | 0.03 |  | 293.34 | **0.000** |
|  | ADHD | -0.52 |  | 0.18 |  | -2.96 | **0.003** |
|  | Autism | -0.13 |  | 0.16 |  | -0.78 | 0.435 |
|  | Dyscalculia | -0.16 |  | 0.07 |  | -2.38 | 0.017 |
|  | Dyslexia | -0.32 |  | 0.05 |  | -6.89 | **0.000** |
| Random intercept | Family variance |  | 0.60 |  | 0.78 |  |  |
|  | SES |  | 0.24 |  | 0.49 |  |  |
|  | Residual |  | 0.64 |  | 0.80 |  |  |

*Note.* *p* < .0125 shown in bold

Table 4. Cont.

| GCSE maths subject test (child-reported) | | | | | | | |
| --- | --- | --- | --- | --- | --- | --- | --- |
| Effects | Variable | *β* | *σ^2^* | *SE* | *SD* | *t* | *p* |
| Fixed | Intercept | 9.13 |  | 0.04 |  | 252.63 | **0.000** |
|  | ADHD | -0.61 |  | 0.20 |  | -3.01 | **0.002** |
|  | Autism | -0.04 |  | 0.19 |  | -0.21 | 0.832 |
|  | Dyscalculia | -0.42 |  | 0.08 |  | -5.35 | **0.000** |
|  | Dyslexia | -0.24 |  | 0.05 |  | -4.46 | **0.000** |
| Random intercept | Family variance |  | 0.91 |  | 0.95 |  |  |
|  | SES |  | 0.33 |  | 0.57 |  |  |
|  | Residual |  | 0.81 |  | 0.90 |  |  |
| Strength and Difficulties Questionnaire peer problems subscale (child-reported) | | | | | | | |
| Effects | Variable | *β* | *σ^2^* | *SE* | *SD* | *t* | *p* |
| Fixed | Intercept | 1.50 |  | 0.02 |  | 71.22 | **0.000** |
|  | ADHD | 0.05 |  | 0.24 |  | 0.19 | 0.850 |
|  | Autism | 0.69 |  | 0.22 |  | 3.12 | **0.001** |
|  | Dyscalculia | 0.00 |  | 0.10 |  | 0.04 | 0.970 |
|  | Dyslexia | 0.18 |  | 0.06 |  | 2.88 | **0.004** |
| Random intercept | Family variance |  | 0.72 |  | 0.85 |  |  |
|  | SES |  | 0.00 |  | 0.00 |  |  |
|  | Residual |  | 1.47 |  | 1.21 |  |  |
| Short Mood and Feeling Questionnaire (child-reported) | | | | | | | |
| Effects | Variable | *β* | *σ^2^* | *SE* | *SD* | *t* | *p* |
| Fixed | Intercept | 3.54 |  | 0.06 |  | 57.12 | **0.000** |
|  | ADHD | -0.28 |  | 0.71 |  | -0.40 | 0.690 |
|  | Autism | 0.83 |  | 0.64 |  | 1.30 | 0.190 |
|  | Dyscalculia | 0.15 |  | 0.28 |  | 0.52 | 0.600 |
|  | Dyslexia | 0.24 |  | 0.18 |  | 1.28 | 0.200 |
| Random intercept | Family variance |  | 6.38 |  | 2.53 |  |  |
|  | SES |  | 0.00 |  | 0.04 |  |  |
|  | Residual |  | 12.38 |  | 3.52 |  |  |
| Strength and Difficulties Questionnaire emotion subscale (child-reported) | | | | | | |  |
| Effects | Variable | *β* | *σ^2^* | *SE* | *SD* | *t* | *p* |
| Fixed | Intercept | 2.72 |  | 0.03 |  | 81.91 | **0.000** |
|  | ADHD | 0.20 |  | 0.36 |  | 0.57 | 0.570 |
|  | Autism | 0.27 |  | 0.33 |  | 0.81 | 0.420 |
|  | Dyscalculia | 0.03 |  | 0.14 |  | 0.22 | 0.830 |
|  | Dyslexia | 0.03 |  | 0.09 |  | 0.37 | 0.710 |
| Random intercept | Family variance |  | 1.57 |  | 1.25 |  |  |
|  | SES |  | 0.03 |  | 0.17 |  |  |
|  | Residual |  | 3.25 |  | 1.80 |  |  |

*Note.* *p* < .0125 shown in bold

Table 4. Cont.

| Childhood Anxiety Sensitivity (child-reported) | | | | | | | |
| --- | --- | --- | --- | --- | --- | --- | --- |
| Effects | Variable | *β* | *σ^2^* | *SE* | *SD* | *t* | *p* |
| Fixed | Intercept | 7.87 |  | 0.08 |  | 94.28 | **0.000** |
|  | ADHD | 0.26 |  | 0.94 |  | 0.28 | 0.780 |
|  | Autism | -0.27 |  | 0.86 |  | -0.43 | 0.670 |
|  | Dyscalculia | 0.28 |  | 0.38 |  | 0.73 | 0.460 |
|  | Dyslexia | 0.09 |  | 0.25 |  | 0.37 | 0.710 |
| Random intercept | Family variance |  | 11.04 |  | 3.32 |  |  |
|  | SES |  | 0.06 |  | 0.25 |  |  |
|  | Residual |  | 22.12 |  | 4.70 |  |  |

*Note.* *p* < .0125 shown in bold

S3. Results of main analyses

Table 5. Summary of RIMM outcomes for each neurodivergent and comparison group at age 12

| Strength and Difficulties Questionnaire hyperactivity/inattention subscale (parent-reported) | | | | | | | |
| --- | --- | --- | --- | --- | --- | --- | --- |
| Effects | Variable | *β* | *σ^2^* | *SE* | *SD* | *t* | *p* |
| Fixed | Intercept | 2.61 |  | 0.03 |  | 84.35 | **0.000** |
|  | ADHD | 0.64 |  | 0.31 |  | 2.06 | 0.039 |
|  | Autism | 1.58 |  | 0.32 |  | 4.92 | **0.000** |
|  | Dyscalculia | 0.41 |  | 0.13 |  | 3.02 | **0.003** |
|  | Dyslexia | 0.48 |  | 0.09 |  | 5.34 | **0.000** |
| Random intercept | Family variance |  | 1.81 |  | 1.34 |  |  |
|  | Residual |  | 3.04 |  | 1.74 |  |  |
| Conners inattention subscale (parent-reported) | | | | | | | |
| Effects | Variable | *β* | *σ^2^* | *SE* | *SD* | *t* | *p* |
| Fixed | Intercept | 5.11 |  | 0.07 |  | 73.30 | **0.000** |
|  | ADHD | 1.93 |  | 0.67 |  | 2.87 | **0.004** |
|  | Autism | 2.44 |  | 0.70 |  | 3.48 | **0.001** |
|  | Dyscalculia | 0.84 |  | 0.29 |  | 2.86 | **0.004** |
|  | Dyslexia | 1.42 |  | 0.20 |  | 7.23 | **0.000** |
| Random intercept | Family variance |  | 10.10 |  | 3.18 |  |  |
|  | Residual |  | 13.70 |  | 3.71 |  |  |
| Conners Hyperactivity/Impulsivity subscale (parent-reported) | | | | | | | |
| Effects | Variable | *β* | *σ^2^* | *SE* | *SD* | *t* | *p* |
| Fixed | Intercept | 3.88 |  | 0.06 |  | 64.54 | **0.000** |
|  | ADHD | 1.59 |  | 0.52 |  | 3.05 | **0.002** |
|  | Autism | 3.12 |  | 0.54 |  | 5.74 | **0.000** |
|  | Dyscalculia | 0.30 |  | 0.22 |  | 1.34 | 0.181 |
|  | Dyslexia | 0.45 |  | 0.15 |  | 3.00 | **0.003** |
| Random intercept | Family variance |  | 9.20 |  | 3.03 |  |  |
|  | Residual |  | 6.99 |  | 2.64 |  |  |
| Childhood Asperger Syndrome Test (parent-reported) | | | | | | | |
| Effects | Variable | *β* | *σ^2^* | *SE* | *SD* | *t* | *p* |
| Fixed | Intercept | 4.56 |  | 0.05 |  | 96.65 | **0.000** |
|  | ADHD | 0.72 |  | 0.44 |  | 1.62 | 0.105 |
|  | Autism | 3.39 |  | 0.46 |  | 7.33 | **0.000** |
|  | Dyscalculia | 0.51 |  | 0.19 |  | 2.65 | **0.008** |
|  | Dyslexia | 0.55 |  | 0.13 |  | 4.31 | **0.000** |
| Random intercept | Family variance |  | 4.97 |  | 2.23 |  |  |
|  | Residual |  | 5.64 |  | 2.38 |  |  |

*Note.* *p* < .0125 shown in bold

Table 5. Cont.

| Reading web test (child-completed) | | | |  |  |  |  |
| --- | --- | --- | --- | --- | --- | --- | --- |
| Effects | Variable | *β* | *σ^2^* | *SE* | *SD* | *t* | *p* |
| Fixed | Intercept | 59.41 |  | 0.19 |  | 310.80 | **0.000** |
|  | ADHD | -2.56 |  | 1.77 |  | -1.44 | 0.148 |
|  | Autism | -1.81 |  | 1.85 |  | -0.98 | 0.329 |
|  | Dyscalculia | -2.11 |  | 0.76 |  | -2.77 | **0.006** |
|  | Dyslexia | -4.41 |  | 0.51 |  | -8.59 | **0.000** |
| Random intercept | Family variance |  | 83.84 |  | 9.16 |  |  |
|  | Residual |  | 88.40 |  | 9.40 |  |  |

*Note.* *p* < .0125 shown in bold

Table 5. Cont.

| Maths web test (child-completed) | | | | | | | |
| --- | --- | --- | --- | --- | --- | --- | --- |
| Effects | Variable | *β* | *σ^2^* | *SE* | *SD* | *t* | *p* |
| Fixed | Intercept | 69.37 |  | 0.19 |  | 359.65 | **0.000** |
|  | ADHD | -4.15 |  | 1.84 |  | -2.25 | 0.024 |
|  | Autism | -3.96 |  | 1.92 |  | -2.06 | 0.039 |
|  | Dyscalculia | -4.80 |  | 0.80 |  | -6.03 | **0.000** |
|  | Dyslexia | -3.53 |  | 0.53 |  | -6.61 | **0.000** |
| Random intercept | Family variance |  | 80.03 |  | 8.95 |  |  |
|  | Residual |  | 100.04 |  | 10.00 |  |  |
| Strength and Difficulties Questionnaire peer problems subscale (parent-reported) | | | | | | | |
| Effects | Variable | *β* | *σ^2^* | *SE* | *SD* | *t* | *p* |
| Fixed | Intercept | 1.02 |  | 0.02 |  | 47.57 | **0.000** |
|  | ADHD | 0.26 |  | 0.20 |  | 1.30 | 0.194 |
|  | Autism | 0.68 |  | 0.21 |  | 3.25 | **0.001** |
|  | Dyscalculia | 0.16 |  | 0.09 |  | 1.84 | 0.065 |
|  | Dyslexia | 0.27 |  | 0.06 |  | 4.60 | **0.000** |
| Random intercept | Family variance |  | 1.04 |  | 1.02 |  |  |
|  | Residual |  | 1.14 |  | 1.07 |  |  |
| Strength and Difficulties Questionnaire peer problems subscale (child-reported) | | | | | | | |
| Effects | Variable | *β* | *σ^2^* | *SE* | *SD* | *t* | *p* |
| Fixed | Intercept | 0.02 |  | 0.02 |  | 60.18 | **0.000** |
|  | ADHD | 0.22 |  | 0.22 |  | 2.32 | 0.020 |
|  | Autism | 0.23 |  | 0.23 |  | 1.10 | 0.270 |
|  | Dyscalculia | 0.10 |  | 0.10 |  | 2.34 | 0.019 |
|  | Dyslexia | 0.06 |  | 0.06 |  | 3.47 | **0.000** |
| Random intercept | Family variance |  | 0.90 |  | 0.95 |  |  |
|  | Residual |  | 1.57 |  | 1.25 |  |  |
| Mood and Feeling Questionnaire (child-reported) | | | | | | | |
| Effects | Variable | *β* | *σ^2^* | *SE* | *SD* | *t* | *p* |
| Fixed | Intercept | 2.20 |  | 0.05 |  | 48.34 | **0.000** |
|  | ADHD | 1.49 |  | 0.46 |  | 3.28 | **0.001** |
|  | Autism | 0.72 |  | 0.48 |  | 1.51 | 0.131 |
|  | Dyscalculia | 0.47 |  | 0.20 |  | 2.34 | 0.019 |
|  | Dyslexia | 0.42 |  | 0.13 |  | 3.16 | **0.001** |
| Random intercept | Family variance |  | 3.85 |  | 1.96 |  |  |
|  | Residual |  | 6.75 |  | 2.60 |  |  |

*Note.* *p* < .0125 shown in bold

Table 5. Cont.

| Strength and Difficulties Questionnaire emotion subscale (child-reported) | | | | | | |  |
| --- | --- | --- | --- | --- | --- | --- | --- |
| Effects | Variable | *β* | *σ^2^* | *SE* | *SD* | *t* | *p* |
| Fixed | Intercept | 2.14 |  | 0.03 |  | 75.53 | **0.000** |
|  | ADHD | 1.02 |  | 0.29 |  | 3.47 | **0.000** |
|  | Autism | 0.34 |  | 0.30 |  | 1.10 | 0.270 |
|  | Dyscalculia | 0.35 |  | 0.13 |  | 2.76 | **0.006** |
|  | Dyslexia | 0.27 |  | 0.09 |  | 3.18 | **0.001** |
| Random intercept | Family variance |  | 1.31 |  | 1.15 |  |  |
|  | Residual |  | 2.96 |  | 1.72 |  |  |

*Note.* *p* < .0125 shown in bold

Table 6. Summary of RIMM outcomes for each neurodivergent and comparison group at age 16

| Strength and Difficulties Questionnaire hyperactivity/inattention subscale (parent-reported) | | | | | | | |
| --- | --- | --- | --- | --- | --- | --- | --- |
| Effects | Variable | *β* | *σ^2^* | *SE* | *SD* | *t* | *p* |
| Fixed | Intercept | 2.14 |  | 0.03 |  | 79.94 | **0.000** |
|  | ADHD | 0.72 |  | 0.26 |  | 2.73 | **0.006** |
|  | Autism | 1.13 |  | 0.28 |  | 4.09 | **0.000** |
|  | Dyscalculia | 0.16 |  | 0.12 |  | 1.37 | 0.172 |
|  | Dyslexia | 0.42 |  | 0.08 |  | 5.46 | **0.000** |
| Random intercept | Family variance |  | 1.38 |  | 1.17 |  |  |
|  | Residual |  | 2.22 |  | 1.49 |  |  |
| Conners inattention subscale (parent-reported) | | | | | | | |
| Effects | Variable | *β* | *σ^2^* | *SE* | *SD* | *t* | *p* |
| Fixed | Intercept | 3.91 |  | 0.07 |  | 58.01 | **0.000** |
|  | ADHD | 2.13 |  | 0.65 |  | 3.29 | **0.001** |
|  | Autism | 2.27 |  | 0.68 |  | 3.37 | **0.000** |
|  | Dyscalculia | 0.63 |  | 0.28 |  | 2.23 | 0.026 |
|  | Dyslexia | 0.87 |  | 0.19 |  | 4.65 | **0.000** |
| Random intercept | Family variance |  | 9.59 |  | 3.10 |  |  |
|  | Residual |  | 12.50 |  | 3.54 |  |  |
| Conners Hyperactivity/Impulsivity subscale (parent-reported) | | | | | | | |
| Effects | Variable | *β* | *σ^2^* | *SE* | *SD* | *t* | *p* |
| Fixed | Intercept | 2.33 |  | 0.05 |  | 48.37 | **0.000** |
|  | ADHD | 1.57 |  | 0.43 |  | 3.65 | **0.000** |
|  | Autism | 1.69 |  | 0.45 |  | 3.77 | **0.000** |
|  | Dyscalculia | 0.14 |  | 0.18 |  | 0.75 | 0.451 |
|  | Dyslexia | 0.30 |  | 0.12 |  | 2.37 | 0.018 |
| Random intercept | Family variance |  | 5.66 |  | 2.38 |  |  |
|  | Residual |  | 4.94 |  | 2.22 |  |  |
| Autism Spectrum Quotient(parent-reported) | | | | | | | |
| Effects | Variable | *β* | *σ^2^* | *SE* | *SD* | *t* | *p* |
| Fixed | Intercept | 23.67 |  | 0.17 |  | 143.57 | **0.000** |
|  | ADHD | -0.09 |  | 1.35 |  | -0.07 | 0.945 |
|  | Autism | 7.11 |  | 1.42 |  | 5.02 | **0.000** |
|  | Dyscalculia | 0.95 |  | 0.57 |  | 2.15 | 0.031 |
|  | Dyslexia | 0.84 |  | 0.39 |  | 1.67 | 0.096 |
| Random intercept | Family variance |  | 73.91 |  | 8.60 |  |  |
|  | Residual |  | 44.36 |  | 6.66 |  |  |

*Note.* *p* < .0125 shown in bold

Table 6. Cont.

| GCSE english subject test (child-reported) | | | | | | |  |
| --- | --- | --- | --- | --- | --- | --- | --- |
| Effects | Variable | *β* | *σ^2^* | *SE* | *SD* | *t* | *p* |
| Fixed | Intercept | 9.07 |  | 0.02 |  | 502.45 | **0.000** |
|  | ADHD | -0.69 |  | 0.16 |  | -4.36 | **0.000** |
|  | Autism | -0.05 |  | 0.17 |  | -0.30 | 0.761 |
|  | Dyscalculia | -0.16 |  | 0.07 |  | -2.42 | 0.016 |
|  | Dyslexia | -0.33 |  | 0.05 |  | -7.24 | **0.000** |
| Random intercept | Family variance |  | 0.82 |  | 0.91 |  |  |
|  | Residual |  | 0.64 |  | 0.80 |  |  |

*Note.* *p* < .0125 shown in bold

Table 6. Cont.

| GCSE maths subject test (child-reported) | | | | | | | |
| --- | --- | --- | --- | --- | --- | --- | --- |
| Effects | Variable | *β* | *σ^2^* | *SE* | *SD* | *t* | *p* |
| Fixed | Intercept | 9.13 |  | 0.02 |  | 427.44 | **0.000** |
|  | ADHD | -0.79 |  | 0.18 |  | -4.36 | **0.000** |
|  | Autism | 0.06 |  | 0.19 |  | 0.31 | 0.751 |
|  | Dyscalculia | -0.39 |  | 0.08 |  | -5.03 | **0.000** |
|  | Dyslexia | -0.25 |  | 0.05 |  | -4.85 | **0.000** |
| Random intercept | Family variance |  | 1.19 |  | 1.09 |  |  |
|  | Residual |  | 0.83 |  | 0.91 |  |  |
| Strength and Difficulties Questionnaire peer problems subscale (child-reported) | | | | | | | |
| Effects | Variable | *β* | *σ^2^* | *SE* | *SD* | *t* | *p* |
| Fixed | Intercept | 1.51 |  | 0.02 |  | 73.80 | **0.000** |
|  | ADHD | 0.02 |  | 0.21 |  | 0.11 | 0.910 |
|  | Autism | 0.67 |  | 0.22 |  | 3.06 | **0.002** |
|  | Dyscalculia | 0.02 |  | 0.09 |  | 0.21 | 0.829 |
|  | Dyslexia | 0.18 |  | 0.06 |  | 3.00 | **0.003** |
| Random intercept | Family variance |  | 0.73 |  | 0.85 |  |  |
|  | Residual |  | 1.47 |  | 1.21 |  |  |
| Short Mood and Feeling Questionnaire (child-reported) | | | | | | | |
| Effects | Variable | *β* | *σ^2^* | *SE* | *SD* | *t* | *p* |
| Fixed | Intercept | 3.29 |  | 0.62 |  | 5.36 | **0.000** |
|  | ADHD | 1.04 |  | 0.85 |  | 1.22 | 0.220 |
|  | Autism | 0.28 |  | 0.62 |  | 0.46 | 0.640 |
|  | Dyscalculia | 0.46 |  | 0.67 |  | 0.69 | 0.490 |
|  | Dyslexia | 0.52 |  | 0.64 |  | 0.81 | 0.420 |
| Random intercept | Family variance |  | 6.48 |  | 2.55 |  |  |
|  | Residual |  | 12.68 |  | 3.56 |  |  |
| Strength and Difficulties Questionnaire emotion subscale (child-reported) | | | | | | | |
| Effects | Variable | *β* | *σ^2^* | *SE* | *SD* | *t* | *p* |
| Fixed | Intercept | 2.68 |  | 0.31 |  | 8.61 | **0.000** |
|  | ADHD | 0.24 |  | 0.43 |  | 0.55 | 0.580 |
|  | Autism | 0.05 |  | 0.31 |  | 0.16 | 0.880 |
|  | Dyscalculia | 0.07 |  | 0.34 |  | 0.20 | 0.840 |
|  | Dyslexia | 0.09 |  | 0.32 |  | 0.27 | 0.790 |
| Random intercept | Family variance |  | 1.58 |  | 1.26 |  |  |
|  | Residual |  | 3.29 |  | 1.82 |  |  |

*Note.* *p* < .0125 shown in bold

Table 6. Cont.

| Childhood Anxiety Sensitivity (child-reported) | | | | | | |  |
| --- | --- | --- | --- | --- | --- | --- | --- |
| Effects | Variable | *β* | *σ^2^* | *SE* | *SD* | *t* | *p* |
| Fixed | Intercept | 7.52 |  | 0.81 |  | 9.30 | **0.000** |
|  | ADHD | -0.10 |  | 1.12 |  | -0.09 | 0.930 |
|  | Autism | 0.36 |  | 0.81 |  | 0.45 | 0.660 |
|  | Dyscalculia | 0.62 |  | 0.88 |  | 0.71 | 0.480 |
|  | Dyslexia | 0.41 |  | 0.84 |  | 0.49 | 0.620 |
| Random intercept | Family variance |  | 11.20 |  | 3.35 |  |  |
|  | Residual |  | 21.90 |  | 4.68 |  |  |

*Note.* *p* < .0125 shown in bold

S4. Longitudinal analysis on repeated measures (group x age interaction)

Table 7 presents the descriptive statistics of the longitudinal analysis derived from the RIMM models. Figures 1 to 5 were plotted using the descriptive data from ages 12 and 16 in Table 7. Detailed statistical outcomes of the longitudinal analysis are provided in Table 8.

Table 7. Descriptives of Longitudinal Analyses of Repeated Measures

|  | ADHD  Age 12 | | ADHD  Age 16 | | Autism  Age 12 | | Autism  Age 16 | |
| --- | --- | --- | --- | --- | --- | --- | --- | --- |
|  | *M* | *SD* | *M* | *SD* | *M* | *SD* | *M* | *SD* |
| ADHD features | |  |  |  |  |  |  |  |
| SDQ HYP/I.P | 2.96 | 2.12 | 2.54 | 1.87 | 4.09 | 2.96 | 2.12 | 2.54 |
| Conners I.P | 6.36 | 4.67 | 5.22 | 4.51 | 7.13 | 6.36 | 4.67 | 5.22 |
| Conners HYP/IMP.P | 5.70 | 3.64 | 2.97 | 3.06 | 6.83 | 5.70 | 3.64 | 2.97 |
| Peer relationship difficulties | | | |  |  |  |  |  |
| SDQ Peer.C | 3.34 | 2.09 | 2.31 | 2.21 | 2.44 | 2.10 | 2.90 | 2.22 |
| Internalizing issues | |  |  |  |  |  |  |  |
| SDQ Emo.C | 1.83 | 1.57 | 1.43 | 1.50 | 1.62 | 1.57 | 2.14 | 1.50 |

*Note.* SDQ HYP/I.P = Strengths and Difficulties Questionnaire hyperactivity/inattention subscale (parent-reported) (score 0 to 10). Conners I.P = Conners inattention subscale (parent-reported) (score 0 to 27), Conners HYP/IMP.P = Conners Hyperactivity/Impulsivity subscale (parent-reported) (score 0 to 27), SDQ.Peer.C = Strength and Difficulties Questionnaire peer problems subscale (child-reported) (score 0 to 10), SDQ.Emo.C = Strength and Difficulties Questionnaire emotion subscale (child-reported) (score 0 to 10).

Table 7. Cont.

|  | Dyscalculia  Age 12 | | Dyscalculia  Age 16 | | Dyslexia  Age 12 | | Dyslexia  Age 16 | | Comparison  Age 12 | | Comparison  Age 16 | |
| --- | --- | --- | --- | --- | --- | --- | --- | --- | --- | --- | --- | --- |
|  | *M* | *SD* | *M* | *SD* | *M* | *M* | *SD* | *SD* | *M* | *SD* | *M* | *SD* |
| ADHD features | |  |  |  |  |  |  |  |  |  |  |  |
| SDQ HYP/I.P | 3.04 | 2.10 | 2.16 | 1.85 | 3.01 | 2.16 | 2.43 | 1.92 | 2.61 | 2.41 | 2.14 | 2.24 |
| Conners I.P | 5.77 | 4.60 | 4.31 | 4.43 | 6.45 | 4.80 | 4.35 | 4.67 | 5.13 | 5.72 | 3.94 | 5.56 |
| Conners HYP/IMP.P | 4.13 | 3.59 | 2.42 | 3.01 | 4.39 | 3.74 | 2.48 | 3.19 | 3.87 | 4.56 | 2.34 | 4.07 |
| Peer relationship difficulties | | | |  |  |  |  |  |  |  |  |  |
| SDQ Peer.C | 2.53 | 2.08 | 2.63 | 2.20 | 2.44 | 2.14 | 2.69 | 2.24 | 2.14 | 2.23 | 2.73 | 2.41 |
| Internalizing issues | |  |  |  |  |  |  |  |  |  |  |  |
| SDQ Emo.C | 1.61 | 1.56 | 1.45 | 1.48 | 1.55 | 1.58 | 1.65 | 1.53 | 1.33 | 1.74 | 1.52 | 1.66 |

Figure 1. Longitudinal rate of change in SDQ HYP/I.
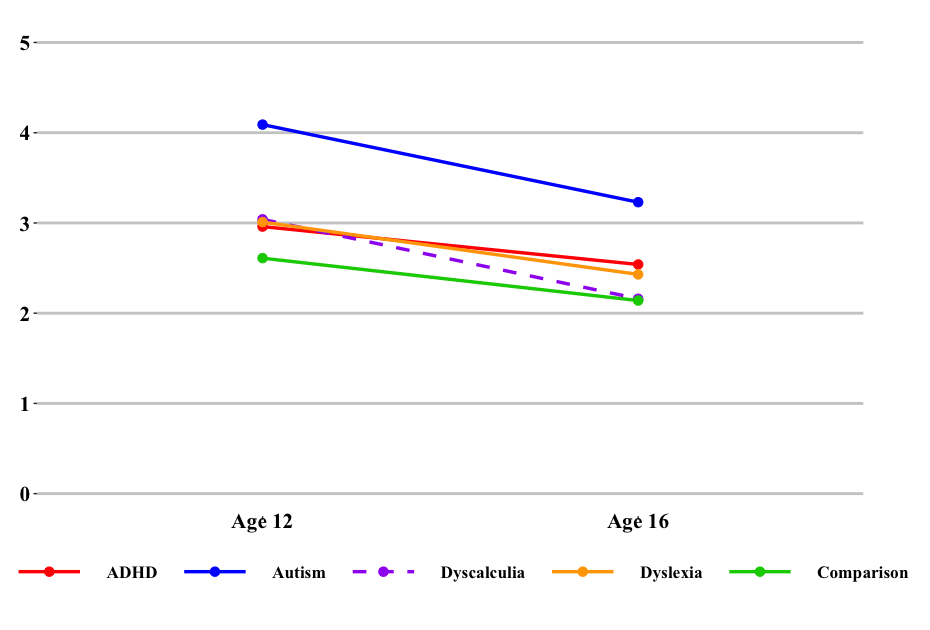
***Note.*** The plot range is set to 0–5 for SDQ subscales and 0–10 for Conners subscales to improve visualization. Dashed lines indicate neurodivergent groups showing significant differences in longitudinal slopes compared to the comparison group.

Figure 2. Longitudinal rate of change in Conners. I.P
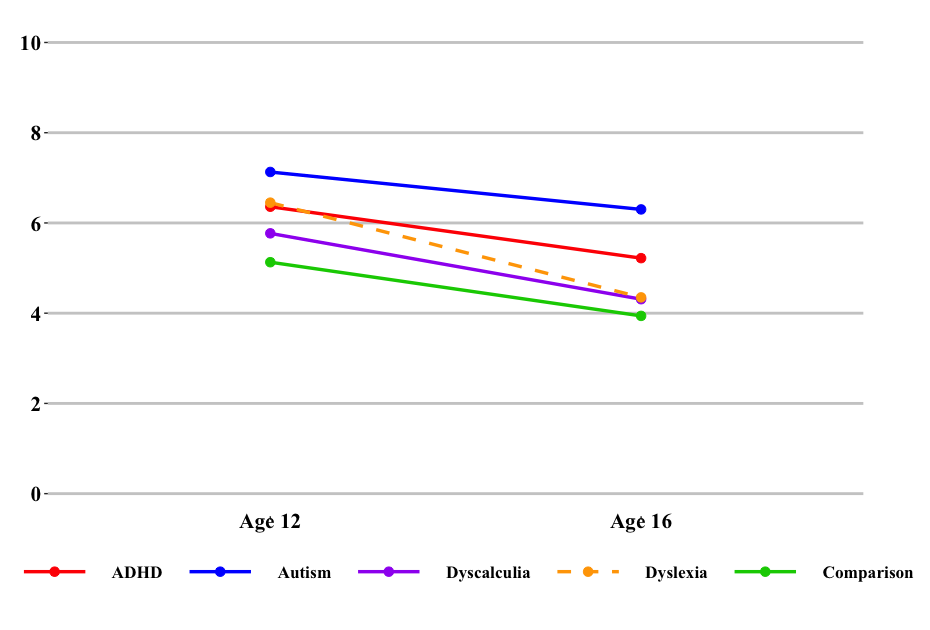


Figure 3. Longitudinal rate of change in Conners.HYP/IMP.P
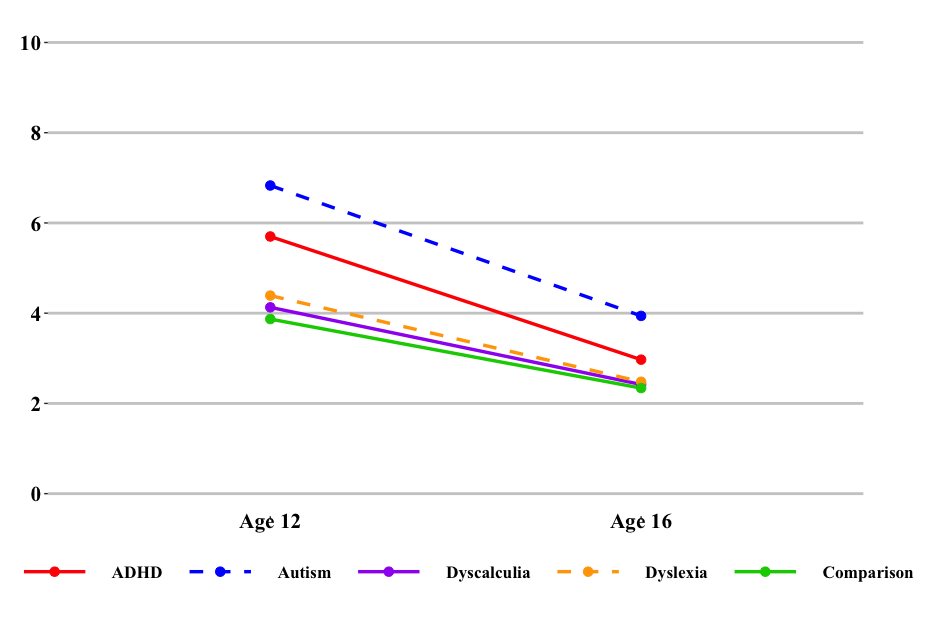


Figure 4. Longitudinal rate of change in SDQ Peer.C


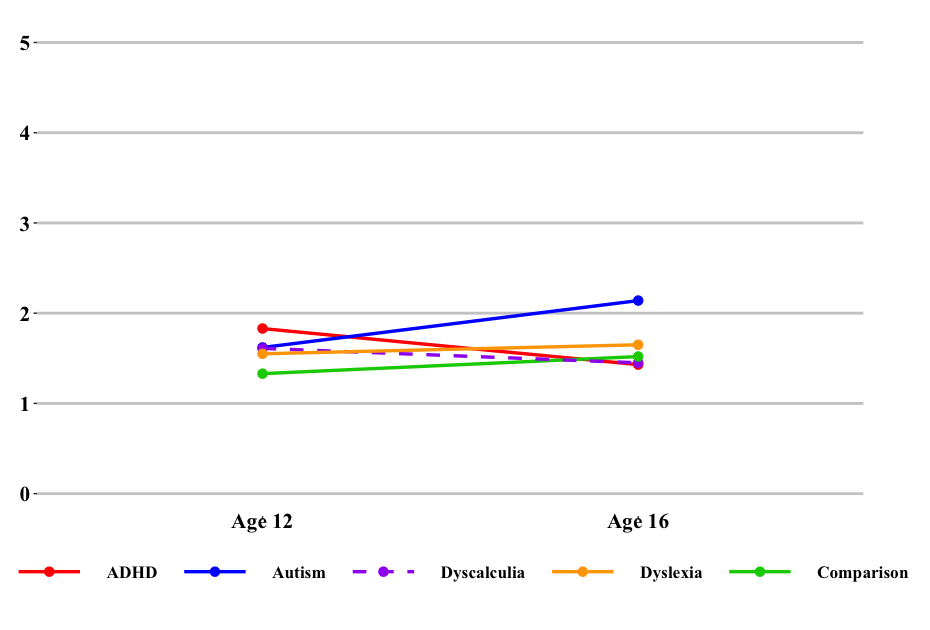


Figure 5. Longitudinal rate of change in SDQ Emo.C


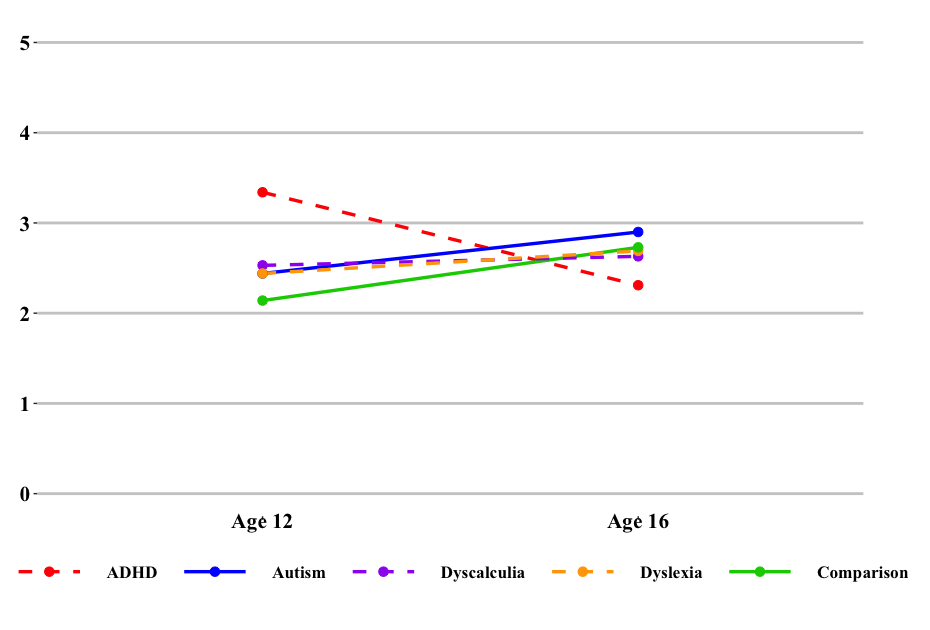


Table 8. Age interaction effect on repeated measures between each neurodivergent to the comparison groups

| Strength and Difficulties Questionnaire hyperactivity/inattention subscale (parent-reported) | | | | | | | | | | |
| --- | --- | --- | --- | --- | --- | --- | --- | --- | --- | --- |
| Effects | Variable | *β* | *σ^2^* | *SE* | | *SD* | | *t* | *p* | |
| Fixed | Intercept | 4.02 |  | 0.10 | |  | | 38.85 | **0.000** | |
|  | Age | -0.12 |  | 0.01 | |  | | -16.79 | **0.000** | |
|  | ADHD | 0.20 |  | 1.16 | |  | | 0.18 | 0.860 | |
|  | Autism | 2.65 |  | 1.21 | |  | | 2.20 | 0.028 | |
|  | Dyscalculia | 1.66 |  | 0.52 | |  | | 3.22 | **0.001** | |
|  | Dyslexia | 0.74 |  | 0.34 | |  | | 2.18 | 0.029 | |
| Interaction | Age: ADHD | 0.01 |  | 0.08 | |  | | 0.15 | 0.879 | |
|  | Age: Autism | -0.10 |  | 0.08 | |  | | -1.19 | 0.235 | |
|  | Age: Dyscalculia | -0.10 |  | 0.04 | |  | | -2.92 | **0.003** | |
|  | Age: Dyslexia | -0.03 |  | 0.02 | |  | | -1.21 | 0.226 | |
| Random intercept | Intercept |  | 1.53 |  | | 1.24 | |  |  | |
|  | Residual |  | 3.20 |  | | 1.79 | |  |  | |
| Conners inattention subscale (parent-reported) | | | | | | | | | | |
| Effects | Variable | *β* | *σ^2^* | | *SE* | | *SD* | *t* | *p* | |
| Fixed | Intercept | 8.69 |  | | 0.23 | |  | 38.00 | **0.000** | |
|  | Age | -0.30 |  | | 0.02 | |  | -19.02 | **0.000** | |
|  | ADHD | 1.10 |  | | 2.55 | |  | 0.43 | 0.666 | |
|  | Autism | 0.92 |  | | 2.65 | |  | 0.35 | 0.729 | |
|  | Dyscalculia | 1.46 |  | | 1.13 | |  | 1.29 | 0.198 | |
|  | Dyslexia | 4.07 |  | | 0.74 | |  | 5.47 | **0.000** | |
| Interaction | Age: ADHD | 0.01 |  | | 0.18 | |  | 0.06 | 0.949 | |
|  | Age: Autism | 0.09 |  | | 0.18 | |  | 0.49 | 0.623 | |
|  | Age: Dyscalculia | -0.07 |  | | 0.08 | |  | -0.86 | 0.388 | |
|  | Age: Dyslexia | -0.23 |  | | 0.05 | |  | -4.43 | **0.000** | |
| Random intercept | Intercept |  | 9.67 | |  | | 3.11 |  |  | |
|  | Residual |  | 14.19 | |  | | 3.77 |  |  |  |
| Conners Hyperactivity/Impulsivity subscale (parent-reported) | | | | | | | | | | |
| Effects | Variable | *β* | *σ^2^* | | *SE* | | *SD* | *t* | *p* | |
| Fixed | Intercept | 8.44 |  | | 0.17 | |  | 49.14 | **0.000** | |
|  | Age | -0.38 |  | | 0.01 | |  | -33.54 | **0.000** | |
|  | ADHD | 5.44 |  | | 1.91 | |  | 2.85 | **0.004** | |
|  | Autism | 7.06 |  | | 1.99 | |  | 3.56 | **0.000** | |
|  | Dyscalculia | 0.82 |  | | 0.85 | |  | 0.97 | 0.330 | |
|  | Dyslexia | 1.69 |  | | 0.56 | |  | 3.04 | **0.002** | |
| Interaction | Age: ADHD | -0.30 |  | | 0.13 | |  | -2.34 | 0.019 | |
|  | Age: Autism | -0.34 |  | | 0.13 | |  | -2.55 | **0.010** | |
|  | Age: Dyscalculia | -0.05 |  | | 0.06 | |  | -0.81 | 0.416 | |
|  | Age: Dyslexia | -0.10 |  | | 0.04 | |  | -2.59 | **0.009** | |
| Random intercept | Intercept |  | 6.08 | |  | | 2.47 |  |  | |
|  | Residual |  | 9.05 | |  | | 3.01 |  |  | |

*Note.* *p* < .0125 shown in bold

Table 8. Cont.

*Note.* *p* < .0125 shown in bold

| Strength and Difficulties Questionnaire peer problems subscale (child-reported) | | | | | | | |  |
| --- | --- | --- | --- | --- | --- | --- | --- | --- |
| Effects | Variable | *β* | *σ^2^* | *SE* | *SD* | *t* | *p* | |
| Fixed | Intercept | 0.76 |  | 0.08 |  | 9.46 | **0.000** | |
|  | Age | 0.05 |  | 0.01 |  | 8.61 | **0.000** | |
|  | ADHD | 2.30 |  | 0.90 |  | 2.56 | **0.010** | |
|  | Autism | -0.68 |  | 0.93 |  | -0.73 | 0.467 | |
|  | Dyscalculia | 1.33 |  | 0.40 |  | 3.34 | **0.000** | |
|  | Dyslexia | 0.47 |  | 0.26 |  | 1.78 | 0.074 | |
| Interaction (Age x Group) | Age: ADHD | -0.15 |  | 0.06 |  | -2.39 | 0.016 | |
|  | Age: Autism | 0.08 |  | 0.06 |  | 1.25 | 0.211 | |
|  | Age: Dyscalculia | -0.09 |  | 0.03 |  | -3.14 | **0.001** | |
|  | Age: Dyslexia | -0.02 |  | 0.02 |  | -1.13 | 0.257 | |
| Random intercept (family variance) | Intercept |  | 0.66 |  | 0.81 |  |  | |
|  | Residual |  | 1.78 |  | 1.34 |  |  | |
| Strength and Difficulties Questionnaire emotion subscale (child-reported) | | | | | |  |  | |
| Effects | Variable | *β* | *σ^2^* | *SE* | *SD* | *t* | *p* | |
| Fixed | Intercept | 0.38 |  | 0.11 |  | 3.46 | **0.000** | |
|  | Age | 0.15 |  | 0.01 |  | 18.80 | **0.000** | |
|  | ADHD | 6.07 |  | 1.25 |  | 4.87 | **0.000** | |
|  | Autism | 0.68 |  | 1.30 |  | 0.52 | 0.600 | |
|  | Dyscalculia | 1.86 |  | 0.55 |  | 3.36 | **0.000** | |
|  | Dyslexia | 1.29 |  | 0.36 |  | 3.56 | **0.000** | |
| Interaction (Age x Group) | Age: ADHD | -0.41 |  | 0.09 |  | -4.59 | **0.000** | |
|  | Age: Autism | -0.03 |  | 0.09 |  | -0.35 | 0.730 | |
|  | Age: Dyscalculia | -0.12 |  | 0.04 |  | -3.12 | **0.001** | |
|  | Age: Dyslexia | -0.08 |  | 0.03 |  | -3.24 | **0.001** | |
| Random intercept (family variance) | Intercept |  | 1.23 |  | 1.11 |  |  | |
|  | Residual |  | 3.10 |  | 1.76 |  |  | |

S5. Unplanned χ^2^ tests of elevated ADHD features and autistic traits

Tables 9 (age 12) and 10 (age 16) present the number of children in each neurodivergent group scoring more than two standard deviations above the mean of the aggregated dataset (N = 7,963) on measures of ADHD and autistic traits, along with the χ² analysis outcomes from pairwise comparisons between each neurodivergent group and the comparison group. Figures 6 to 8 were generated using data from Tables 9 and 10.

At 12 years, all four neurodivergent groups had a significantly higher proportion of children with elevated inattention scores (ADHD 15%; autism 14%; dyscalculia 9%; dyslexia 10%) than the comparison group (4%). A greater proportion of children in the ADHD (26%), autism (20%), and dyslexia (8%) groups had clinically elevated hyperactivity/impulsivity scores compared to the comparison group (4%), but this was not observed in the dyscalculia group (6%). All four neurodivergent groups had a significantly greater proportion of children with elevated autistic traits (ADHD 19%; autism 24%; dyscalculia 10%; dyslexia 12%) than the comparison group (3%). At 16 years, the proportion of elevated inattention scores remained significantly higher in the ADHD (24%), autism (16%), and dyslexia (7%) groups than in the comparison group (5%), but not in the dyscalculia group (6%). Elevated hyperactivity/impulsivity scores were significantly higher across all neurodivergent groups (ADHD 19%; autism 14%; dyscalculia 8%; dyslexia 7%) compared to the comparison group (5%). Clinically elevated autistic traits were significantly higher only in the autism group (24%) than in the comparison group (3%), while the percentages in the other groups (ADHD 4%; dyscalculia 4%; dyslexia: 3%) did not differ significantly from the comparison group.

Figure 6.


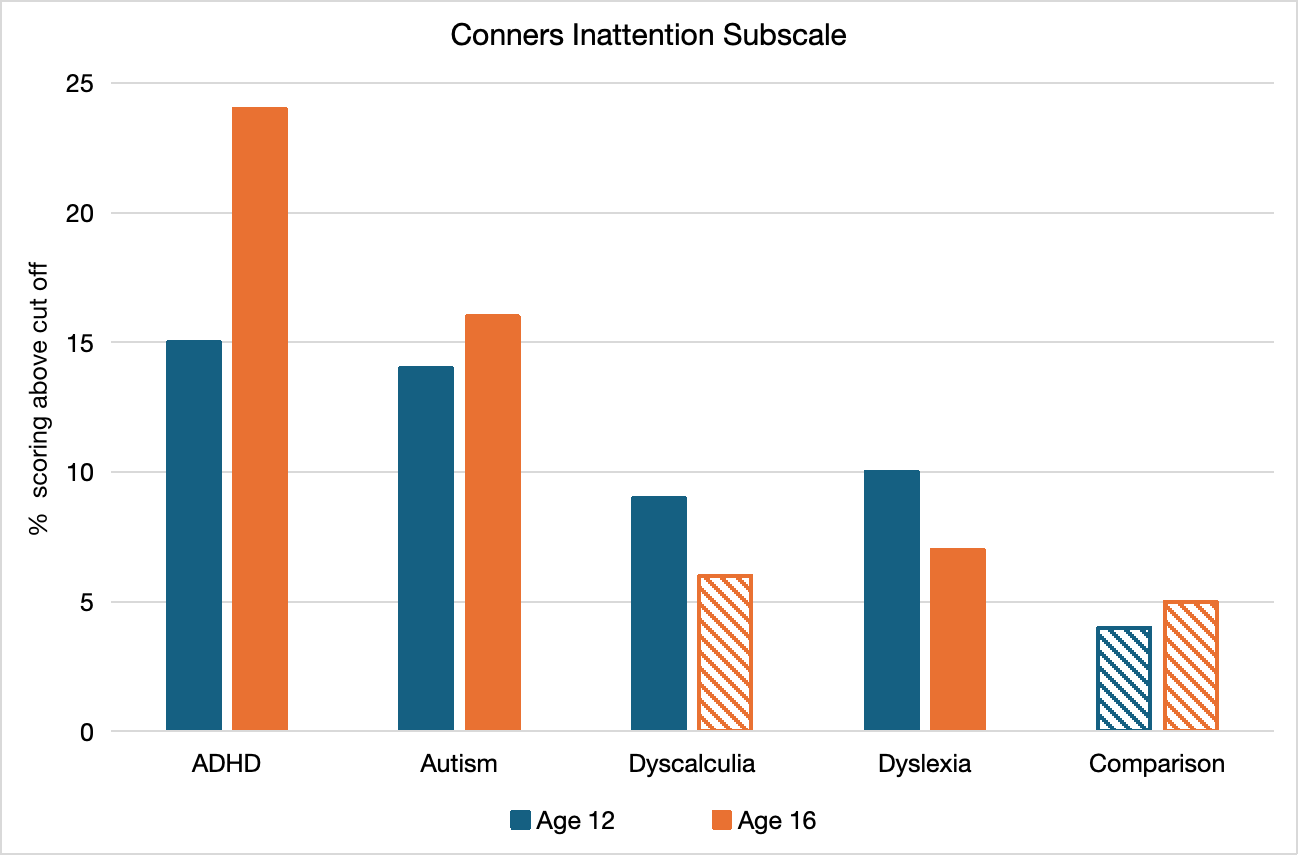


*Note.* Solid bars indicate a significant difference in the percentage of individuals scoring above the cut-off between the neurodivergent group and the comparison group; dashed bars denote no significant difference.

Figure 7.


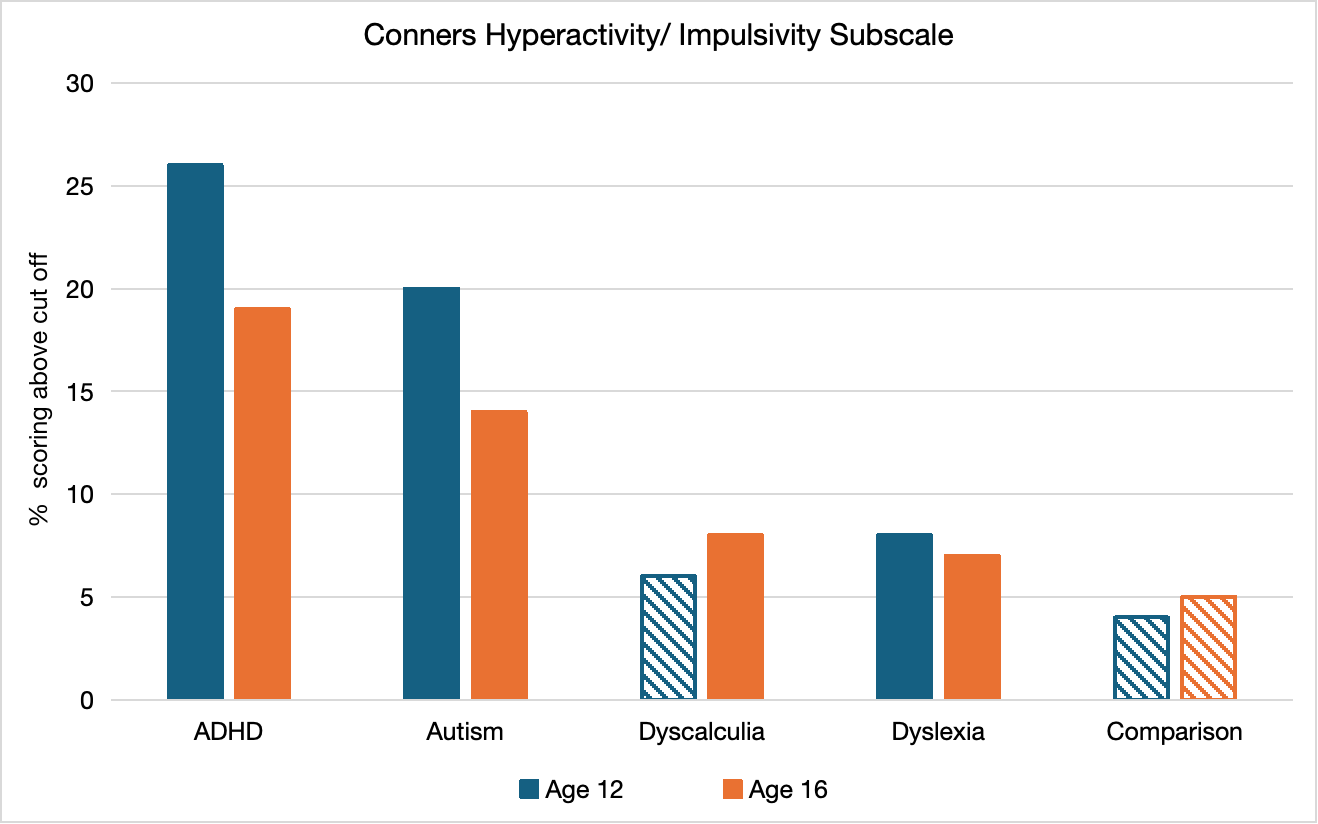


Figure 8.


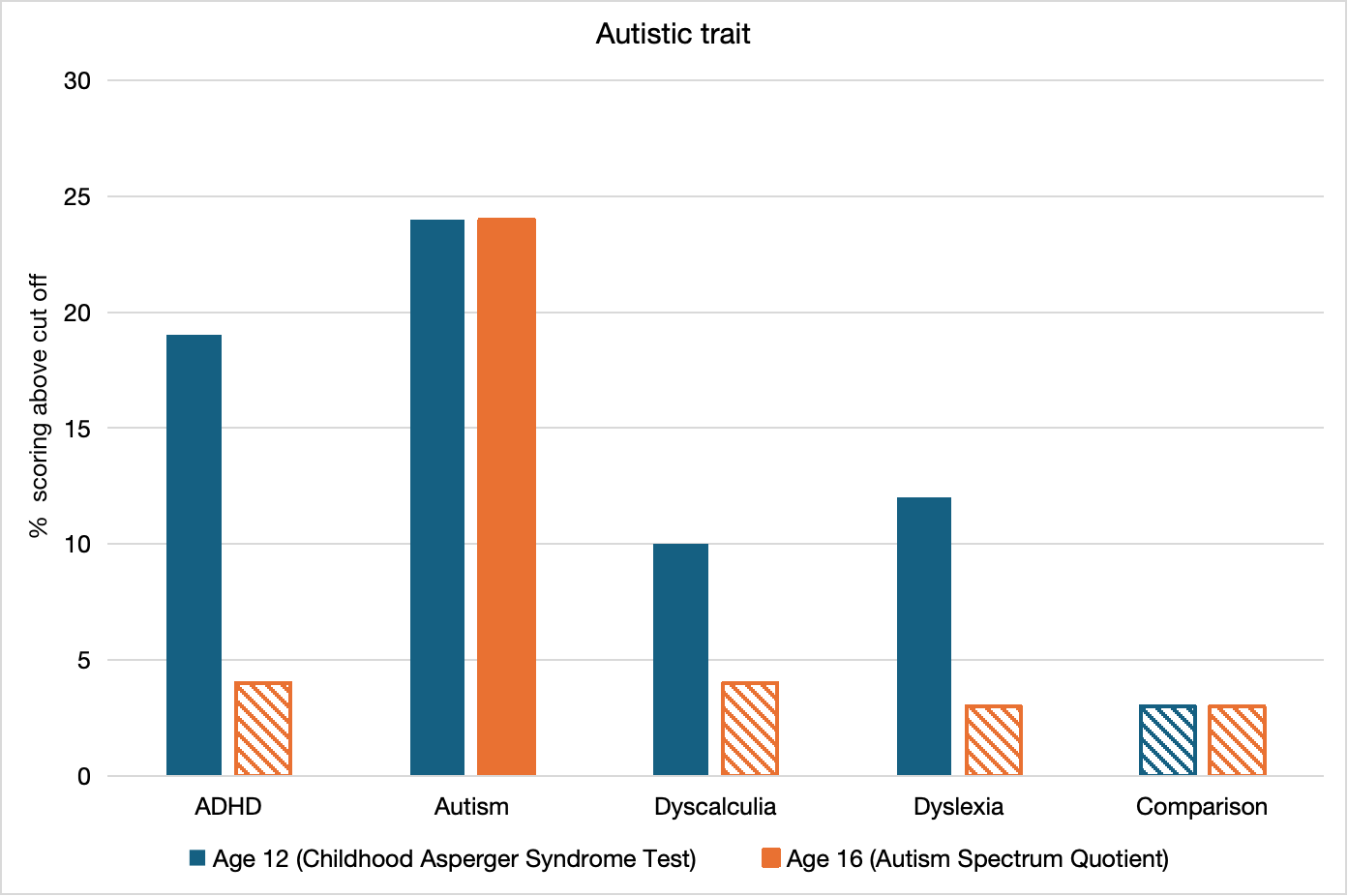


|  | Conners inattention scale | | |  |
| --- | --- | --- | --- | --- |
|  | % of children who score equal or above the cut-off | % of children who score below the cut-off | χ^2^ statistics | *p*-value vs. comparison group |
| ADHD (*n* = 54) | 15% | 85% | 15.27 | **0.000** |
| Autism (*n* = 50) | 14% | 86% | 12.11 | **0.000** |
| Dyscalculia (*n* = 282) | 9% | 91% | 12.78 | **0.000** |
| Dyslexia (*n* = 695) | 10% | 90% | 52.80 | **0.000** |
| Comparison group  (*n* = 6882) | 4% | 96% | - | - |
|  | Conners hyperactivity/ impulsivity scale | | | |
|  | % of children who score equal or above the cut-off | % of children who score below the cut-off | χ^2^ statistics | *p*-value vs. comparison group |
| ADHD (*n* = 54) | 26% | 74% | 66.05 | **0.000** |
| Autism (*n* = 50) | 20% | 80% | 33.10 | **0.000** |
| Dyscalculia (*n* = 282) | 6% | 94% | 2.17 | 0.141 |
| Dyslexia (*n* = 695) | 8% | 92% | 24.48 | **0.000** |
| Comparison group  (*n* = 6882) | 4% | 96% | - | - |
|  | Childhood Asperger Syndrome Test | | | |
|  | % of children who score equal or above the cut-off | % of children who score below the calculated cut-off | χ^2^ statistics | *p*-value vs. comparison group |
| ADHD (*n* = 54) | 19% | 81% | 15.62 | **0.000** |
| Autism (*n* = 50) | 24% | 76% | 29.49 | **0.000** |
| Dyscalculia (*n* = 282) | 10% | 90% | 7.19 | **0.007** |
| Dyslexia (*n* = 695) | 12% | 88% | 43.68 | **0.000** |
| Comparison group  (*n* = 6882) | 3% | 97% | - | - |

Table 9. Results of χ^2^ analysis at age 12

*Note. p <* .05 shown in bold

Table 10. Results of χ^2^ analysis at age 16

|  | Conners inattention scale | | |  |
| --- | --- | --- | --- | --- |
|  | % of children who score equal or above to the calculated cut-off | % of children who score below to the calculated cut-off | χ^2^ statistics | *p*-value vs. comparison group |
| ADHD (*n* = 54) | 24% | 76% | 41.30 | **0.000** |
| Autism (*n* = 50) | 16% | 84% | 13.01 | **0.003** |
| Dyscalculia (*n* = 282) | 6% | 97% | 0.98 | 0.322 |
| Dyslexia (*n* = 695) | 7% | 93% | 7.86 | **0.005** |
| Comparison group  (*n* = 6882) | 5% | 95% | - | - |
|  | Conners hyperactivity/ impulsivity scale | | | |
|  | % of children who score equal or above to the calculated cut-off | % of children who score below to the calculated cut-off | χ^2^ statistics | *p*-value vs. comparison group |
| ADHD (*n* = 54) | 19% | 81% | 22.74 | **0.000** |
| Autism (*n* = 50) | 14% | 86% | 9.65 | **0.002** |
| Dyscalculia (*n* = 282) | 8% | 96% | 5.13 | **0.024** |
| Dyslexia (*n* = 695) | 7% | 93% | 9.80 | **0.002** |
| Comparison group  (*n* = 6882) | 5% | 95% | - | - |
|  | Autism Spectrum Quotient | | | |
|  | % of children who score equal or above to the calculated cut-off | % of children who score below to the calculated cut-off | χ^2^ statistics | *p*-value vs. comparison group |
| ADHD (*n* = 54) | 4% | 96% | 0.17 | 0.685 |
| Autism (*n* = 50) | 24% | 76% | 78.18 | **0.000** |
| Dyscalculia (*n* = 282) | 4% | 96% | 1.77 | 0.184 |
| Dyslexia (*n* = 695) | 3% | 97% | 1.00 | 0.316 |
| Comparison group  (*n* = 6882) | 3% | 97% | - | - |

*Note. p <* .05 shown in bold
